# Exposure to benzotriazoles and benzothiazoles in Czech male population and its associations with biomarkers of liver function, serum lipids and oxidative stress

**DOI:** 10.1007/s00420-024-02059-x

**Published:** 2024-03-28

**Authors:** Nina Pálešová, Lucie Bláhová, Tomáš Janoš, Katarína Řiháčková, Aleš Pindur, Ludmila Šebejová, Pavel Čupr

**Affiliations:** 1 RECETOX, Faculty of Science, Masaryk University, Kamenice 753/5, 625 00, Brno, Czech Republic; 2 Faculty of Sports Studies, Masaryk University, Kamenice 753/5, 625 00, Brno, Czech Republic; 3 Training Centre of Fire Rescue Service, General Directorate of Fire Rescue Service of the Czech Republic, Ministry of the Interior, Trnkova 85, 628 00, Brno, Czech Republic

**Keywords:** Benzotriazole, Benzothiazole, Human biomonitoring, Liver function, Serum lipids, Oxidative stress

## Abstract

**Introduction:**

Benzotriazoles and benzothiazoles (BTs) are high-production volume chemicals as well as widely distributed emerging pollutants with potential health risk. However, information about human exposure to BTs and associated health outcomes is limited.

**Objective:**

We aimed to characterise exposure to BTs among Czech men, including possible occupational exposure among firefighters, its predictors, and its associations with liver function, serum lipids and oxidative stress.

**Methods:**

165 participants (including 110 firefighters) provided urine and blood samples that were used to quantify the urinary levels of 8 BTs (high-performance liquid chromatography-tandem mass spectrometry), and 4 liver enzymes, cholesterol, low-density lipoprotein, and 8-hydroxy-2’-deoxyguanosine. Linear regression was used to assess associations with population characteristics and biomarkers of liver function, serum lipids and oxidative stress. Regression models were adjusted for potential confounding variables and false discovery rate procedure was applied to account for multiplicity.

**Results:**

The BTs ranged from undetected up to 46.8 ng/mL. 2-hydroxy-benzothiazole was the most predominant compound (detection frequency 83%; median 1.95 ng/mL). 1-methyl-benzotriazole (1M-BTR) was measured in human samples for the first time, with a detection frequency 77% and median 1.75 ng/mL. Professional firefighters had lower urinary 1M-BTR compared to non-firefighters. Urinary 1M-BTR was associated with levels of γ-glutamyl transferase (β = − 17.54%; 95% CI: − 26.127, − 7.962).

**Conclusion:**

This is the first study to investigate BT exposure in Central Europe, including potentially exposed firefighters. The findings showed a high prevalence of BTs in the study population, the relevance of 1M-BTR as a new biomarker of exposure, and an urgent need for further research into associated adverse health outcomes.

**Graphical abstract:**

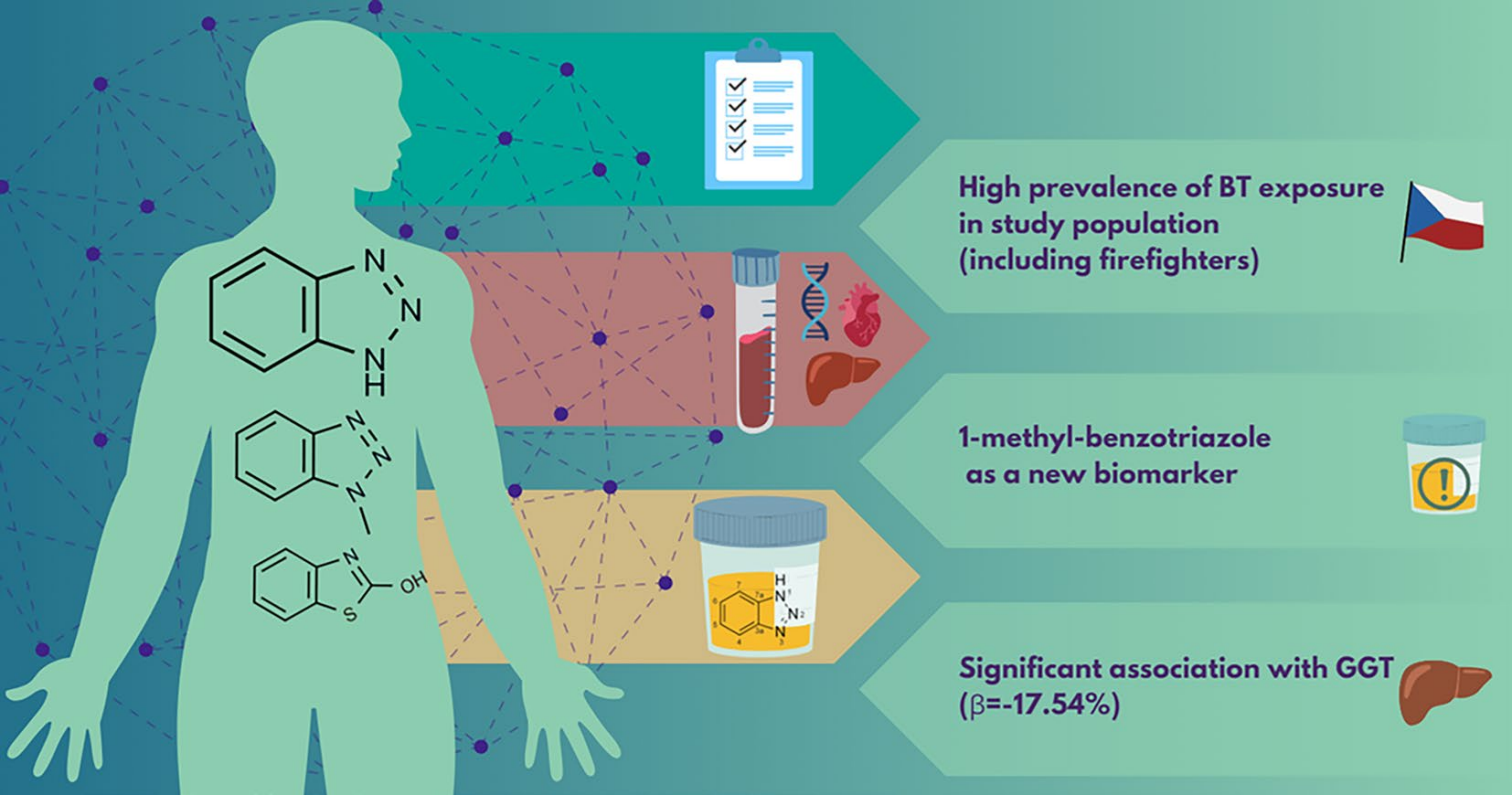

**Supplementary Information:**

The online version contains supplementary material available at 10.1007/s00420-024-02059-x.

## Introduction

Benzotriazoles (BTRs) and benzothiazoles (BTHs) are nitrogen-containing benzo-heterocyclic compounds (collectively called BTs) classified as high-production volume chemicals and emerging pollutants, currently applied in a variety of industrial activities and household products (e.g., dishwasher detergents) (Liao et al. 2018; Shi et al. 2019). BTs are extensively used as corrosion inhibitors for copper and its alloys, UV-stabilisers, flame retardants, de-icing and antifogging agents (Kokalj et al. 2011; Montesdeoca-Esponda et al. 2020; Naccarato et al. 2014), biocides (in the paper and leather industries), and vulcanization accelerators in rubber production (Liao et al. 2018). Due to their wide use along with poor removal efficiency in conventional wastewater treatment plants (WWTP), BTs have been detected in environmental matrices, including virtually all types of water (Loos et al. 2010; Neuwald et al. 2022; Shi et al. 2019; Wang et al. 2023), indoor air and dust (Wan et al. 2016; Wang et al. 2013; Xue et al. 2017), soil (Speltini et al. 2016) and biosolids (Lai et al. 2014). Naturally, BTs have been detected in human urine (Asimakopoulos et al. 2012, 2013), amniotic fluid (Li et al. 2018), and adipose tissue (Wang et al. 2015), and the estimated main exposure routes are via diet (including drinking water) (Castro et al. 2023b; Janna et al. 2011; LeFevre et al. 2017), air pollution inhalation (Maceira et al. 2018; Wan et al. 2016; Wang et al. 2013), and dermal contact (Avagyan et al. 2015; Liu et al. 2017). Firefighters are of particular concern due to potential occupational exposure from aqueous film forming foams (AFFFs), in which BTRs are used as anticorrosive agents (Ateia et al. 2023; Norman & Regina 1993; Titaley et al. 2022), and from smoke, which can contain both BTRs and BTHs due to their widespread application (e.g., as flame retardants) (Bonner et al. 2023; Poutasse et al. 2020; Zhang et al. 2020). However, there is still lack of data on, and understanding of human BT exposure and its links with health outcomes. No complex human biomonitoring study has yet been carried out in Czechia nor anywhere in central or eastern Europe yet. There are few studies from Europe (Asimakopoulos et al. 2012, 2013), however, majority of the studies have focused on the exposure of pregnant women in Asia due to potential prenatal exposure (Cao et al. 2023; Chen et al. 2020; Li et al. 2017, 2018; Zhou et al. 2018, 2020). Extrapolation of these data to other populations can introduce bias due to regional differences in lifestyle factors, as well as legislation and policies concerning BT exposure.

Scientific literature covers various types of adverse effects of BTs, such as endocrine disruption, neurotoxicity, and developmental toxicity, all reported in in vitro and in vivo animal studies (Liao et al. 2018; Shi et al. 2019). Adverse effects in pregnant women have also recently been addressed in some epidemiological studies (Cao et al. 2023; Zhou et al. 2020), revealing that BT exposure during pregnancy was associated with adverse maternal and infant health outcomes. Hepatotoxicity and oxidative stress have also been reported in experimental organisms after exposure to BTs (Duan et al. 2017; Liang et al. 2017). Alteration of liver proteome and the hypertrophy of hepatocytes (probably induced by oxidative stress and cell apoptosis) were observed in fish models after BT exposure (Duan et al. 2017; Kim et al. 2022; Liang et al. 2017). Activation of the liver peroxisome proliferator-activated receptor α (PPARα), cytochrome P450, and UDP-glucuronosyltransferase (UDP-GT) and glutathione-S-transferase were reported in rats after the administration of BTs (Hirata-Koizumi et al. 2009; Seo et al. 2000). Hence, BT exposure might be a potential risk factor with respect to the development of liver injury also in humans. However, such associations have not yet been investigated in an epidemiological study. This taken together with recent epidemics of metabolic diseases, including increased rates of such diseases in firefighters (Soteriades et al. 2011), indicates an urgent need to identify modifiable risk factors (e.g., environmental pollutants) and focus on effective prevention strategies.

Information on exposure levels in the male population or in occupationally exposed individuals is very limited. This is the first study of its kind in central Europe, which, investigated BT urinary concentrations in 165 men from Czechia, with a special focus on potential occupational exposure among firefighters. In addition, potential predictors of urinary BTs as well as associations of urinary BTs with liver function, serum lipid, and oxidative stress biomarkers were examined. This paper provides insights into new extraction method for BTs in human urine and should increase the understanding of BT exposure patterns among men from central Europe as well as implications for their liver health.

## Materials and methods

### Study population

The present study included 165 participants of the CELSPAC-FIREexpo study, a collaborative research project with the aim of assessing firefighters´ exposure to contaminants during firefighting (FF) and FF training, and also of determining chemical and biochemical biomarkers of exposure and related biological responses. The study population is described in detail in a previous work (Řiháčková et al. 2023). Briefly, all participants were physically active men from Czechia, who were 18–35-year-old non-smokers with no chronic diseases. Participants were divided into 3 study sub-cohorts according to their relationship with FF: newly recruited firefighters before any professional training for active participation in responses to incidents (“NEW”; n = 58), professional firefighters actively participating in responses to incidents (“PROF”; n = 52), and a control group of non-firefighters recruited at the Faculty of Sport, Masaryk University, Brno (Czech Republic) (“CTRL”; n = 55). Upon inclusion in the study, all participants answered questionnaires and provided morning void urine for the analyses of BTs and oxidative stress biomarker, and a fasting blood sample for the analyses of liver function and serum lipid biomarkers. A subset of randomly selected participants (n = 20) from CTRL (n = 10) and NEW (n = 10) also provided a second sample of urine 10 weeks later. Information regarding the questionnaires and the transportation and storage of samples is available in Supplementary Information (SI) (section Sample collection and storage and Table S1). The study was approved by the ELSPAC Ethics Committee in 2019, and all participants gave their written informed consent.

### Assessment of exposure

#### Sample pre-treatment

A list of chemical compounds and reagents used is available in SI (section Chemicals and reagents). Frequently used BTs and their potential metabolites were identified on the basis of a literature search and a total number of six BTRs (1-H-benzotriazole [1H-BTR], 4-OH-benzotriazole [4OH-BTR], 1-methyl-benzotriazole [1M-BTR], 4-methyl-benzotriazole [4M-BTR], 5-methyl-benzotriazole [5M-BTR] and xylyltriazole [XTR]) and two BTHs (2-hydoxy-benzothiazole [2OH-BTH] and 2-amino-benzothiazole [2NH2-BTH]) were analysed. The isomers 4M-BTR and 5M-BTR were expressed as their sum (4/5M-BTR). The sum of free and conjugated forms in urine was determined following a procedure reported in a previous study (Bláhová et al. 2023) with modifications. Urine samples were thawed and vortexed, and 500 µL of urine sample was introduced into a 2 mL plastic tube. 10 µL of a mixture of isotopically labelled internal standards (*d4*-1H-BTR and *d5*-atrazine) were added to achieve the concentration in samples 10 and 2 ng/mL, respectively. Next, the samples were spiked with *β*-glucuronidase (500 µL, 1000 U/mL in 1 M CH_3_COONH_4_, from *Helix pomatia*), vortexed, and incubated overnight (37 °C, 170 rpm) to release free forms via enzymatic de-conjugation. The enzymatic reaction was stopped by freezing at − 80 °C (6 h). The samples were then freeze-dried and extracted with 500 µL of isopropanol. Different types of *β*-glucuronidase (*E.coli*) and extraction solvents (based on literature search: acetonitrile (Bláhová et al. 2023), acetonitrile:dichloromethane (1:1) (Asimakopoulos et al. 2012), and methyl tert-butyl ether:ethyl acetate (5:1) (Li et al. 2017)) were also tested. A better de-conjugation effect for BTs and lower concentrations of analytes in blanks (the contamination of blanks) were observed for *β*-glucuronidase from *Helix pomatia* when compared to *E.coli* (data not shown). All tested solvents resulted in similar recoveries. Insoluble particles were removed by centrifugation (12,000 rcf, 10 min, 10 °C); the clear supernatants were evaporated to dryness and then reconstituted in 10% methanol (v/v). Possible residual particles in final extracts were removed using microspin filters (0.2 µm, cellulose acetate, Fisher Scientific). Filtrates were stored in glass inserts at − 20 °C until instrumental analyses.

#### Instrumental analysis

A Waters Acquity LC chromatograph (Waters, Manchester, U.K.) coupled with a Xevo TQ-S quadrupole mass spectrometer (Waters Manchester, U.K.) (LC–MS/MS) operated in positive electrospray ionization (ESI) mode was used for the determination of BTs. The ionization parameters were as follows: capillary voltage, 0.84 kV; source temperature and desolvation temperature, 150 and 500 °C, respectively; cone gas flow, 150 L/h; desolvation gas flow, 600 L/h; and collision gas flow, 0.15 mL/min. The cone voltage and collision energy were optimized for each analyte. The MRM transitions are shown in Supplementary Information (Table S2). A BEH C18 (100 × 2.1 mm; 1.7 μm) analytical column (Waters) kept at 40 °C was used for chromatographic separation. Acidified (0.1% formic acid) water (A) and acetonitrile (B) were used as mobile phases with the following gradient—10% B at 0–1 min, 10% to 40% B at 1–3 min, 40% to 80% B at 3–7 min, and 80% of B kept for 1 min followed by 2 min of equilibration to the initial conditions (10% B). The flow rate was 0.35 mL/min, and 9 μL was injected for the analyses. Data were processed by MassLynxTM software (Waters, Manchester, U.K.). The concentrations of analytes were corrected for the content of internal standards.

Urine creatinine levels and specific gravity were determined for the adjustment of urinary BT levels. Creatinine levels were determined by LC–MS/MS using a modified procedure described by Derezińsky and collective (Dereziński et al. 2016). Urine specific gravity (SG) was measured using a handheld PAL-10 S refractometer (Atago, Japan). Formulas used for the adjustments are presented in the Supplementary Information (Table S3).

#### Quality control and quality assurance

After the extraction procedure, two procedural blanks and one spiked sample (water spiked with target compounds, 5 ng/mL) were included in each analysis batch. Quality control samples were analysed after every 25 urine samples and repeatability was found to be acceptable (RSD ≤ 11.2%). The mean recoveries of analytes in spiked samples were in the range of 94.4–116.3% (Table S4).

The method limits of quantification (MLOQ) were calculated as 3 times the standard deviation (SD) of the blanks (N = 8) and for analytes that were not detected in the procedural blanks, a signal-to-noise ratio (S/N) > 10 was used as a criterion for the estimation of MLOQ. The MLOQs are summarized in Table S2. For BTs detected in blanks, the measured concentrations were corrected by subtracting their respective median blank concentrations (Table S4).

### Assessment of liver function, serum lipid, and oxidative stress biomarkers

The levels of the enzymes alanine aminotransferase (ALT, in μkat/L), alkaline phosphatase (ALP, in μkat/L), aspartate aminotransferase (AST, in μkat/L), γ-glutamyl transferase (GGT, in μkat/L) and total bilirubin (TBIL, in μmol/L) in blood serum were considered as markers of liver function. Total serum cholesterol (CHOL, mmol/L) and low-density lipoprotein (LDL, mmol/L) were considered as indicators of blood serum lipids. Both liver and lipid biomarkers were measured spectrophotometrically with an Alinity c instrument (©Abbott, Illinois, USA).

8-hydroxy-2’-deoxyguanosine (8OHdG), a biomarker of oxidative stress, was measured in urine by LC–MS/MS following the method described in detail previously (Bláhová et al. 2023). Briefly, thawed urine samples (500 µL) were spiked with internal standard (15N5-8-hydroxy-2′-deoxyguanosine), vortexed, and then lyophilized. After extraction with isopropanol, supernatants were evaporated to dryness and redissolved in 0.1% formic acid (v/v), and the final extracts were stored at -20 °C until LC–MS/MS analysis.

### Statistical analysis

Detection frequencies (DF), means, and selected percentiles (25th, 50th and 75th) were calculated to describe the distribution profiles of SG-adjusted and creatinine-adjusted urinary BTs. SG-adjusted urine BT levels were used for further statistical analyses (Sauvé et al. 2015). Creatinine-adjusted and unadjusted levels were calculated to allow easy comparison with other published studies. Only compounds with DF higher than 50% were imputed based on maximum likelihood multiple estimations dependent on observed values and their distribution (log-normal) (Lubin et al. 2004), and then included in subsequent analyses. The intraclass correlation coefficient (ICC), ratio of between-individual variance to the sum of between- and within-individual variances, was employed to estimate the temporal variability of urinary BTs using the subset of participants (n = 20), who provided a second urine sample 10 weeks after the first sample. Selected percentiles (25th, 50th and 75th) were calculated for biomarkers of liver function, serum lipids, and oxidative stress. SG-adjusted and imputed urinary BTs and SG-adjusted 8OHdG, as well as liver function, serum lipid and oxidative stress biomarkers measured in serum were log2 transformed to address skewness and improve the normality of the distribution.

Spearman´s correlation coefficients were computed to assess pair-wise correlations between individual BTs, demographic characteristics of the study population (age, BMI, FF career length and contact with FF foams) and biomarkers of liver function, serum lipids and oxidative stress. Statistical differences between the sub-cohorts were investigated by ANOVA/Kruskal–Wallis ANOVA with Tukey/Wilcox post hoc tests. Then, the data were standardized for the interquartile range (to reduce the influence of outliers) and used in linear regression models to examine the associations of urinary BTs with demographic characteristics of the study population, such as age (in years), BMI (in kg/m2), former smoking (yes/no), study sub-cohort (CTRL/NEW/PROF), sampling season (spring/summer/winter/autumn), and contact with FF foams in the last year (never/one time/two or more times). Next, associations between urinary BTs (explanatory variables) and biomarkers of liver function, serum lipids, and oxidative stress (dependent variables) were assessed using multiple linear regression models. Minimal sufficient adjustment set of confounding factors included in multiple linear regression models was identified on the basis of a priori knowledge, directed acyclic graph (DAG, Fig. S1) (Shrier & Platt 2008) and results from linear regression models. Firstly, a basic model (Model 1) was constructed and adjusted for age (in years), BMI (in kg/m2), and former smoking (yes/no). Additional potentially confounding variables were included in the second model (Model 2) – sampling season (spring/summer/winter/autumn) and study sub-cohort (CTRL/NEW/PROF). To reduce false positive findings due to the multiplicity of statistical tests, the false discovery rate (FDR) procedure was applied (Benjamini & Yekutieli 2005).

Sensitivity analyses were performed to test the robustness of the obtained results. Considering the potential correlations among urinary BTs which have similar sources, we constructed the Multiple-BTs model by including all BTs in the multiple linear regression models simultaneously in order to estimate associations with liver function, serum lipid and oxidative stress biomarkers after controlling for all BTs. To assess the sensitivity of the obtained results to the urinary dilution adjustment method, linear regression analyses were performed again with data adjusted for creatinine instead of specific gravity.

### Estimated daily intake

The daily intake of BTs was estimated based on urinary concentrations of ∑8 BTs and a simple steady-state kinetic model. To calculate ∑8 BTs, imputed and SG-adjusted values were used for analytes with a detection frequency higher than 50%, while for the rest of the BTs, concentrations below MLOQ were substituted with the value MLOQ/square root of 2. The values of all 8 BTs were then summed for each participant. The estimation of daily intake was undertaken using the following formula derived from an equation used in previous studies (Katsikantami et al. 2019; Šulc et al. 2022):

$$EDI \,\left( {\mu g/kg /day} \right) = \frac{{c_U \,\left( {\mu g/L} \right) \times V_{UO} \,(L /day)}}{{F_{UE} \times b.w.\,(kg)}}$$

where EDI is Estimated Daily Intake, c_U_ is the SG-adjusted concentration of BTs in urine, V_UO_ is the urine output volume (1.7 L/day (Perucca et al. 2007)), F_UE_ is the urinary excretion factor, and b.w. is the body weight of the participant. However, the pharmacokinetics of BTs is not well known; hence, 3 theoretical excretion scenarios with different F_UE_ were considered: A) a worst-case scenario, in which only 10% of daily intake is excreted through urine (F_UE_ = 0.10); B) a medium scenario, in which 50% of daily intake is excreted via urine (F_UE_ = 0.50); and lastly C) a best-case scenario, in which 90% of daily intake is excreted via urine (F_UE_ = 0.90). All statistical analyses were performed using Rstudio version 4.2.3 (RStudio Team 2020).

## Results

### Characteristics of the study population

Population characteristics and the levels of selected biomarkers are shown in Table 1. All participants were physically active men, non-smoking men between the ages of 18 to 35 years; the mean age was 26.4 ± 4.3 years. The mean BMI was 25.8 ± 2.7 and 13% of participants reported former smoking. Regarding the study sub-cohorts, PROF participants were slightly older compared to NEW and CTRL participants and the mean of the firefighting career length was 4.6 ± 3.4 years. The PROF sub-cohort also had the highest proportion of participants who had been in contact with firefighting foams two or more times in the previous year. PROF had statistically lower levels of ALP. Lower levels of GGT and higher levels of 8OHdG were observed for the CTRL sub-cohort. PROF had statistically higher levels of CHOL and LDL.

**Table 1:** Population characteristics and liver function, serum lipid and oxidative stress biomarkers for CELSPAC-FIREexpo study participants

Characteristics	Overall study population	NEW	PROF	CTRL
	*n* = 165	*n* = 58	*n* = 52	*n* = 55
	Mean ± SD			
Age (years)	26.4 ± 4.3	25.0 ± 3.6	28.4 ± 3.6*	25.9 ± 4.8
BMI	25.8 ± 2.7	26.3 ± 2.8^+^	26.1 ± 2.4	24.9 ± 2.7
FF career length	1.68 ± 2.8	0.67 ± 0.66	4.58 ± 3.4	0 ± 0
Former smoking (yes)	13.0%	12.0%	19.0%	7.4%
Contact with FF foams in the last year				
Never	65.5%	69.5%	25.0%	98.2%
One time	22.4%	25.4%	40.4%	1.8%
Two or more times	12.1%	0.0%	34.6%	0.0%

Biomarkers	Median (25–75th percentile)			
TBIL (µmol/L)	13.1 (9–17)	14.0 (9.23–17)	13.0 (9–17)	13.0 (9.5–18.5)
ALP (µkat/L)	1.19 (1.02–1.4)	1.21 (1.04–1.42)	1.10* (0.98–1.24)	1.25 (1.06–1.53)
ALT (µkat/L)	0.43 (0.34–0.56)	0.42 (0.34–0.53)	0.48 (0.38–0.64)	0.42 (0.33–0.54)
AST (µkat/L)	0.47 (0.4–0.62)	0.46 (0.4–0.54)	0.45 (0.38–0.61)	0.55 (0.45–0.68)
GGT (µkat/L)	0.34 (0.25–0.44)	0.35 (0.26–0.43)	0.39 (0.29–0.53)	0.29* (0.22–0.34)
CHOL (mmol/L)	4.5 (4.1–5.2)	4.5 (3.9–4.8)	4.9* (4.4–5.4)	4.4 (3.8–5.1)
LDL (mmol/L)	2.8 (2.4–3.3)	2.6 (2.3–3.1)	3.2* (2.7–3.7)	2.8 (2.2–3.3)
8OHdG (SG) (µg/L)	4.90 (3.83–6.59)	4.58 (3.52–6.13)	4.56 (3.79–6.02)	5.82* (4.29–8)

“ * “ means statistically different from other sub-cohorts. “ + “ means statistically different from controls

The Spearman correlation matrix is available in Supplementary Information (Fig. S2). Briefly, levels of ALT, GGT, CHOL, and LDL were positively correlated with BMI and length of FF career. ALP, AST, and 8OHdG were negatively correlated with FF career length. Age was negatively correlated with TBIL and ALP, but positively correlated with ALT, CHOL, and LDL. ALP, ALT, and GGT were negatively correlated with 1H-BTR. Moreover, GGT was negatively correlated also with 1M-BTR. 1H-BTR, 1M-BTR, and 2OH-BTH did not significantly correlate with each other. 1M-BTR was negatively correlated with FF career length, contact with FF foams, and former smoking.

### Urinary concentrations of BTs

Urinary concentrations of BTs were measured in 165 samples. DFs and the levels of SG-adjusted urinary BTs are displayed in Table 2. Creatinine-adjusted and unadjusted values are available in Table S5 and S6. DFs ranged from 1.8% up to 83% and only three BTs (1H-BTR, 1M-BTR, and 2OH-BTR) were detected in over 50% of urine samples. The most frequently detected compound was 2OH-BTH (83.0%), followed by 1M-BTR (77.0%) and 1H-BTR (50.3%). These 3 BTs were included in subsequent analyses. 2OH-BTH was also the most abundant, with a median concentration of 1.95 ng/mL, followed by 1M-BTR, with a median of 1.79 ng/mL. The highest maximum concentration (46.8 ng/mL) was observed for 1M-BTR. The ICCs for urinary 1H-BTR, 1M-BTR, and 2OH-BTH were 0.54, 0.48, and 0.46, respectively (Table S7).

**Table 2:** Detection frequencies (DF, %) and distribution profiles of SG-adjusted urinary BT concentrations (ng/mL) (*n* = 165)

Analytes	DF (%)	Mean	SD	Min	Percentile			Max
					25th	50th	75th	
1H-BTR	50.3	0.83	1.63	< MLOQ	< MLOQ	0.34	0.95	13.2
4OH-BTR	17.0	0.37	1.42	< MLOQ	< MLOQ	< MLOQ	< MLOQ	12.1
1 M-BTR	77.0	3.71	6.32	< MLOQ	0.17	1.79	3.80	46.8
4/5 M-BTR	1.80	< MLOQ		< MLOQ	< MLOQ	< MLOQ	< MLOQ	1.95
XTR	4.80	< MLOQ		< MLOQ	< MLOQ	< MLOQ	< MLOQ	0.43
2OH-BTH	83.0	2.52	2.65	< MLOQ	0.99	1.95	3.40	21.0
2NH2-BTH	3.00	< MLOQ		< MLOQ	< MLOQ	< MLOQ	< MLOQ	1.82

**Fig. 1:**
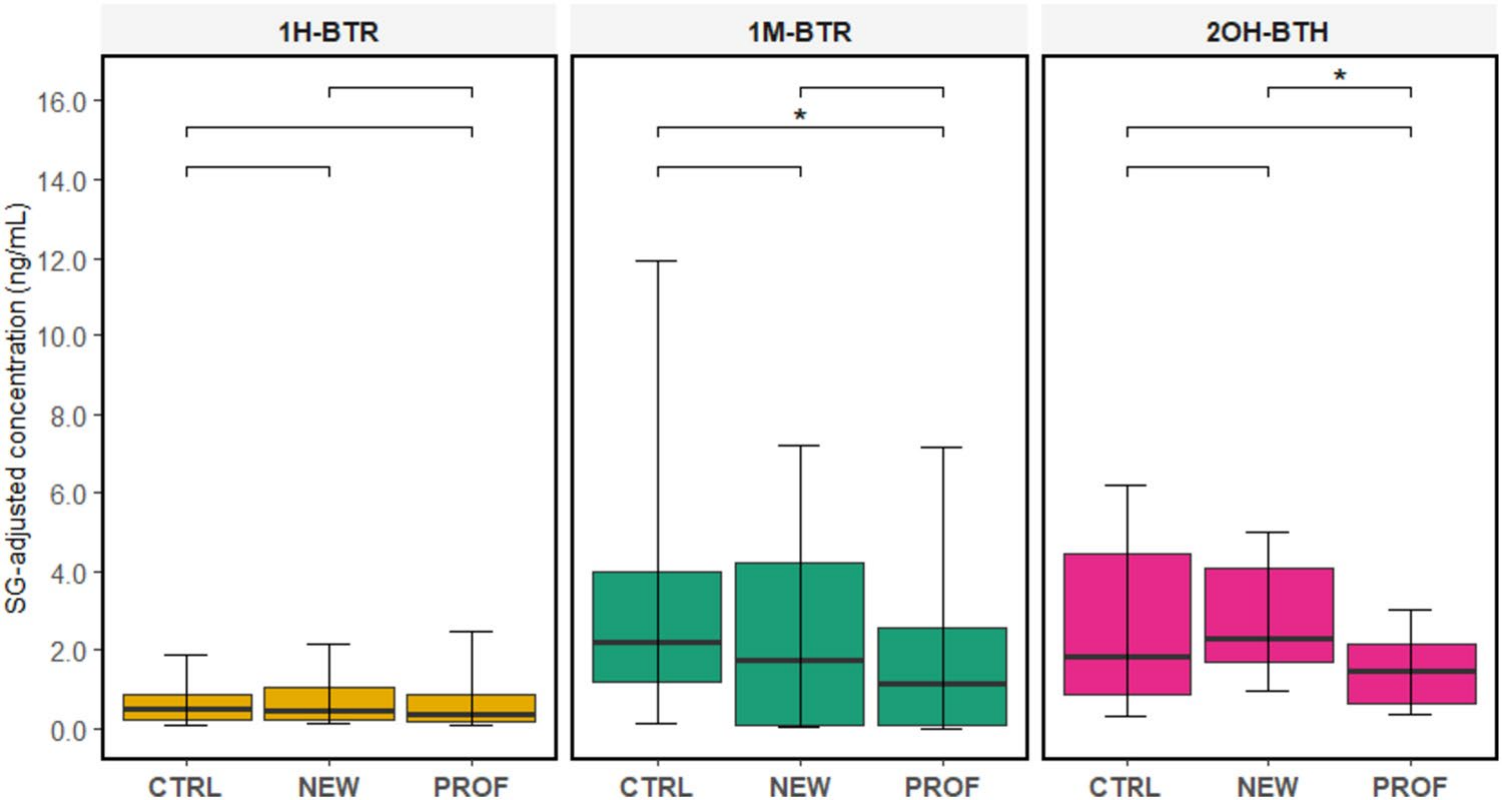
Levels of SG-adjusted urinary BTs (1H-BTR, 1M-BTR, and 2OH-BTH) in the study sub-cohorts. Boxplots display medians (horizontal lines), first and third quartiles (boxes), and 10th and 90th percentiles (whiskers). “*” refers to a statistically significant difference (*p* < 0.05)

Sub-cohort-specific data are displayed in Fig. 1 and Table S8. Significantly higher concentrations of 1M-BTR were observed in CTRL compared to PROF. In the case of 2OH-BTH, NEW had significantly higher levels compared to PROF. Results from linear regression models (Table 3) showed that sub-cohort and contact with AFFFs were predictors of urinary 1M-BTR (p < 0.05), which is in line with descriptive statistics. PROF and NEW tended to have lower urinary levels of 1M-BTR compared to CTRL. Participants who had been in contact with AFFFs two or more times in the previous year tended to have lower levels of 1M-BTR.

**Table 3:** Associations between SG-adjusted concentrations of BTs in urine and population characteristics

Characteristics	1H-BTR		1 M-BTR		2OH-BTH	
	β	*p* value	β	*p* value	β	*p* value
Age	− 4.2	0.685	4.3	0.648	− 1.8	0.858
BMI	− 12.0	0.146	− 0.3	0.970	− 6.6	0.427
Sub-cohort						
CTRL	Reference		Reference		Reference	
NEW	2.3	0.881	− 24.0	0.039*	24.7	0.134
PROF	− 6.0	0.696	− 29.5	0.011*	− 21.8	0.104
Length of FF career	− 4.6	0.305	− 5.5	0.157	− 3.6	0.419
Contact with FF foams in the last year						
Never	Reference		Reference		Reference	
One time	6.6	0.682	− 21.7	0.069	0.3	0.984
Two or more times	− 7.5	0.698	− 30.9	0.032*	− 5.0	0.794
Sampling season						
Autumn	Reference		Reference		Reference	
Spring	18.8	0.282	2.7	0.851	− 10.7	0.477
Summer	7.0	0.660	29.3	0.058	− 14.1	0.317
Winter	− 46.2	0.057	− 2.0	0.942	− 27.6	0.315
Former smoking						
No	Reference		Reference		Reference	
Yes	7.6	0.701	− 30.7	0.027*	− 13.2	0.449

β-coefficient refers to the relative change (%) in urinary BTs for every unit increase/change in population characteristics

*statistical significance (*p* < 0.05)

Former smoking was negatively associated with 1M-BTR. Sampling season was not a significant predictor of urinary BTs. Age, BMI, and length of FF were not significantly associated with any urinary concentrations of BTs (p > 0.05). The results were consistent with sensitivity analysis (Table S9).

### Associations of urinary BTs with liver function, serum lipid, and oxidative stress biomarkers

**Fig. 2:**
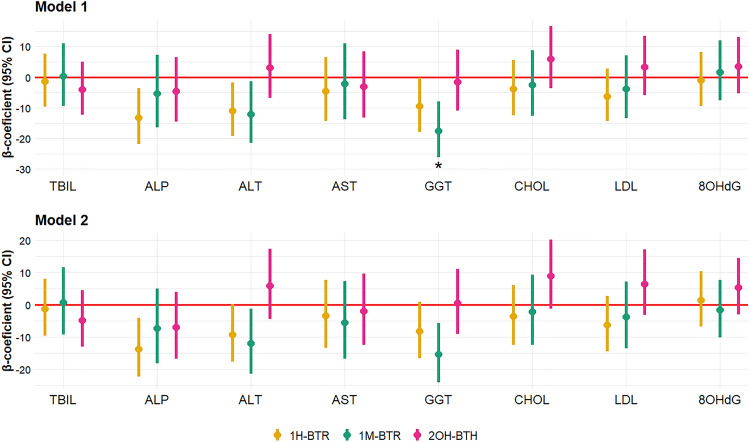
Adjusted β-coefficients and 95% confidence intervals (CI) between the SG-adjusted urinary levels of BTs and the set of biomarkers from the multiple linear regression model (*n* = 165). Estimates are expressed as the percentage change in the median of the biomarker upon doubling of the BT urinary concentration. Model 1 was adjusted for age, BMI, and former smoking; Model 2 was adjusted for age, BMI, former smoking, study sub-cohort, and sampling season. “*” refers to statistical significance after FDR correction

Multiple linear regression models showed some negative associations with liver enzymes across both diversely adjusted models and the results are presented in Fig. 2 and Tables S10 and S11. Results are expressed as the percentage change in the biomarker upon the doubling of the SG-adjusted concentration of BTs in urine. Urinary 1H-BTR was negatively associated with ALP, ALT, and GGT in Model 1. In Model 2, 1H-BTR remained significantly associated only with ALP (− 13.7%, 95% CI: − 22.4%, − 4.1%). The observed associations of 1H-BTR with ALT and GGT in Model 1 were probably caused by associations with sampling season and study sub-cohort, which were included in Model 2 as confounding variables. 1M-BTR was also associated with ALT and GGT, and these associations were significant in both models. Relative changes − 12.0% (95% CI: − 21.5%, − 1.4%) and − 15.5% (95% CI: − 24.2%, − 5.7%) per doubling of urinary 1M-BTR were observed in Model 2 for ALT and GGT, respectively. These three associations (1H-BTR and ALP, 1M-BTR and ALT, and 1M-BTR and GGT) were considered the most robust ones because they were observed also in sensitivity analyses (Table S12 and S13). However, after FDR correction for the multiplicity of statistical tests, only the negative association of 1M-BTR with GGT in Model 1 remained significant.

### Estimated daily intake

The median values of EDI for the sum of 8 BTs based on a simple steady-state model and best-case, medium, and worst-case scenarios were 0.14, 0.25, and 1.30 µg/kg of b.w./day, respectively (Table 4). The best-case scenario ranged from 0.03 to 1.4 µg/kg of b.w./day, the medium scenario from 0.05 up to 2.4 µg/kg of b.w./day, and the worst-case scenario from 0.25 up to 12 µg/kg of b.w./day. No statistically significant differences in EDIs were observed between the sub-cohorts (Table S14).

**Table 4:** Estimated daily intakes (µg/kg of body weight/day) for the sum of 8 BTs for 3 theoretical toxicokinetic scenarios. F_UE_ – urine excretion factor, SD – standard deviation

	F_UE_	Mean	SD	Percentile		
				25th	50th	75th
Best-case scenario	0.9	0.19	0.19	0.09	0.14	0.23
Medium scenario	0.5	0.35	0.33	0.15	0.25	0.42
Worst-case scenario	0.1	1.70	1.70	0.77	1.30	2.10

## Discussion

To the best of our knowledge this is the first study focused on characterization of exposure and predictors of urinary BTs in Czech as well as central European male population. 3 out of 8 analysed BTs had detection rate higher than 50%, which suggest prevalent exposure to BTs among study population. The studied compounds were not significantly correlated with each other implying different sources of exposure.

The observed exposure levels were comparable with those found in other currently available studies (the same order of magnitude) (Asimakopoulos et al. 2012, 2013; Cao et al. 2023; Chen et al. 2020; Li et al. 2018; Zhou et al. 2018, 2020). There is only one European study population with measured urinary BT levels (Asimakopoulos et al. 2012, 2013). Geometric means of unadjusted urine concentrations of 1H-BTR and 2OH-BTH in the Greek male sub-population (n = 50) were 1.05 and 1.77 ng/mL, respectively, which is comparable with the results from our study (Table S6). However, the DFs were markedly lower in the case of the Greek male sub-population—16% and 8% for 1H-BTR and 2OH-BTH, respectively (Asimakopoulos et al. 2012). Inconsistency in DF was probably caused by different limits of quantification, which were lower in our study, and hence, made our study more sensitive. A recent study from Wuhan (China) reported urinary BT levels in pregnant women (Zhou et al. 2018). The detection frequencies of 1H-BTR and 2OH-BTH were similar (60% and 81%, respectively), but the median values were lower compared to our study (0.1 and 0.28 ng/mL, respectively), more profoundly in the case of 2OH-BTH (almost 7 times lower). The maximum values for 1H-BTR and 2OH-BTH were higher (36 and 160 ng/mL, respectively), suggesting the presence of extreme values in the Chinese population. Higher DF compared to our study were observed for XTR, 4/5 M-BTR and 2NH2-BTH (57–66%). They reported median levels below our MLOQs, which was probably the main reason for the observed differences (Zhou et al. 2018). A similar pattern of BT urine profiles was observed in three other Chinese studies of pregnant women from Wuhan (Cao et al. 2023; Chen et al. 2020; Zhou et al. 2020). In general, besides various limits of quantification arising from analytical procedures and varying sample sizes, also regional exposure differences, demographic characteristics of the population (especially sex), lifestyle factors and physiological parameters need to be considered. Moreover, relatively low ICCs observed in this study (< 0.6) are in line with previous studies (Cao et al. 2023; Chen et al. 2020; Zhou et al. 2018) and imply that urinary BTs are highly variable in time.

In contrast to previous work, we reported the detection of 1M-BTR in human urine for the first time. There is very little information available about this compound regarding its industrial use or its toxicological potential; however, it has been detected in the environment, including rivers (Loos et al. 2010) and local WWTP effluent (Fialová et al. 2023). Benzotriazoles are ubiquitous in the environment, hence, possible products of microbial biodegradation need to be considered along with parental compounds in the assessment of human exposure. Biotransformation rates, BT metabolites, as well as specific pathways vary substantially under different redox conditions. In both anaerobic and aerobic conditions, 5M-BTR was found to be transformed into 1H-BTR, which was found to be transformed into 1M-BTR (Alotaibi et al. 2015; Huntscha et al. 2014; Liu et al. 2011). Besides potential human exposure to 1M-BTR through drinking water, dietary exposure should be considered as a potential exposure pathway due to the contamination of aquatic ecosystems (and exposure via the consumption of fish and seafood) (Castro et al. 2023a) and the use of various types of water bodies (including treated wastewater and sludge from WWTP) for the irrigation of agricultural fields, which can additionally result in plant up-take (Kodešová et al. 2023). For these reasons and in light of the obtained results in our study, we highly recommend the use of urinary 1M-BTR as a new potential biomarker of exposure to benzotriazoles in future biomonitoring studies along with currently used portfolio of BT biomarkers.

AFFFs, firefighting foams used for extinguishing flammable liquid fires (e.g., from petroleum-based fuels), have recently become a cause of significant concern due to their high content of per- and polyfluoroalkyl surfactants (PFAS) (Řiháčková et al. 2023). Besides PFAS, they contain also benzotriazole corrosion inhibitors, and, hence, represent a potential occupational source of BTR exposure for firefighters (Ateia et al. 2023; Norman & Regina 1993; Titaley et al. 2022). The results suggest that in this study, AFFFs were probably not a significant exposure source for firefighters, probably due to the low frequency of AFFF use during responses to incidents. However, short-term increases in BT levels, followed by fast metabolization and excretion shortly after exposure to AFFFs cannot be ruled out due to unknown pharmacokinetics. Such fluctuations have already been reported in firefighters with respect to urinary polycyclic aromatic hydrocarbons (PAHs) after contact with fire (Řiháčková et al. 2023). Moreover, we hypothesise that the observed differences in urinary BTs between the sub-cohorts might also be due to exposure to other chemicals. Firefighting is one of the most hazardous occupations combining extreme physical and psychological demands with exposure to both high temperatures and a complex mixture of hazardous pollutants (Barros et al. 2021; Trowbridge et al. 2020). As reported in our previous study, both PROF and NEW had significantly higher levels of PFAS and PAHs in their serum and urine samples compared to the control sub-cohort (Řiháčková et al. 2023). We assume such a load of chemicals due to occupation can exploit detoxifying systems, which can result in slower metabolization (and excretion) of xenobiotics compared to individuals with lower overall chemical exposure. Regarding negative associations with former smoking, we assume that this observation might be biased by the low abundance of former smokers in our study cohort (12.7%), which could affect the statistical power. Sampling season was not a significant predictor of urinary BTs although it has been reported as such previously (Zhou et al. 2018). Seasonal variability of BT exposure occurs due to their use as aircraft de-icing and anti-icing fluids (ADAFs), which are massively used during the cold season creating contamination hotspots around airports (Olds et al. 2022; Seeland et al. 2012). No strong air traffic in the South Moravian region might be a reason for the lack of seasonal variability in this study.

A previous experimental study on rare minnows (*Gobiocypris rarus*) demonstrated adverse effects of BT exposure on liver proteome (the alteration of 26 proteins related mainly to xenobiotic clearance, oxidative stress response, apoptosis, and translation) as well as histopathological changes in liver tissue, including the hypertrophy of hepatocytes (Liang et al. 2017). Increased levels of antioxidant enzymes such as glutathione-S-transferase (GST), catalase (CAT), and superoxide dismutase (SOD), and the increased expression of liver-specific fatty acid binding protein were observed in medaka (*Oryzias latipes*) and zebrafish (*Danio rerio*) (Duan et al. 2017; Kim et al. 2022). BTHs also markedly increased the activities of hepatic cytochrome P450 monooxygenases, UDP-glucuronosyltransferase, and GST in male Sprague–Dawley rats after 5 days of exposure (Seo et al. 2000). However, there is still little known about the effects on human health.

In this study we observed negative association between 1M-BTR and GGT. GGT (γ-glutamyl transferase) is a glycosylated protein that catalyses the transfer of the γ-glutamyl moiety from glutathione (GSH) or glutathione-conjugates to acceptors like amino acids and dipeptides. It is critical for maintaining GSH and cysteine homeostasis (Barrios et al. 2001; Chang Jean et al. 2002; Zhang & Forman 2009). Elevated serum GGT activity is an adaptive response against oxidative and toxic stress, and it has been conventionally considered a clinical marker of liver diseases (Zhang & Forman 2009). A positive association between GGT and levels of hepatotoxic pollutants in humans have been reported frequently (Costello et al. 2022; Farzan et al. 2016; Omoike et al. 2021). Besides GGT being a marker of toxicant-related oxidative stress, it has also been suggested that serum GGT may indicate chronic low-level inflammation (Farzan et al. 2016). Moreover, GGT is inversely associated with serum concentrations of antioxidants such as α-carotene, β-cryptoxanthin, zeaxanthin, lycopene, and vitamin C (Lim et al. 2004). Hence, we speculate that serum GGT levels might represent the overall body capacity for the metabolization and excretion of BTs (and xenobiotics in general), which is more effective in individuals with lower serum GGT compared to individuals with higher serum GGT, which might indicate unfavourable conditions such as chronic inflammation or a lack of antioxidants. Despite the potential of BTs to cause hepatotoxicity and oxidative stress observed from experimental studies, no further associations were detected, which might be due to the lack of sensitivity of the chosen biomarkers and/or the relatively low exposure levels. More sensitive molecular biomarkers need to be investigated in the future in order to understand hidden mechanisms and assess associated liver health risks. Moreover, sex-related differences in the adverse effects of BTs, which have been discussed and reported previously (Cao et al. 2023; Liang et al. 2014; Zhou et al. 2020), might also be a reason for the lack of significant associations in our male study population.

Due to mostly polar structures, BTs and their potential metabolites are likely to be directly excreted from urine. However, little is known about BT pharmacokinetics. Major sources of BT exposure for the general population are considered to be drinking water (Janna et al. 2011; LeFevre et al. 2017; van Leerdam et al. 2009; Wang et al. 2023), indoor and outdoor dust (Maceira et al. 2020; Wang et al. 2013), and diet (Castro et al. 2023b; LeFevre et al. 2017). Additional exposure through the skin from clothing containing benzotriazoles has also been reported (Liu et al. 2017). Estimated daily intakes calculated for the abovementioned exposure sources are reported in picograms up to tens of nanograms per kilogram of body weight (Castro et al. 2023b; Maceira et al. 2020; Wang et al. 2013, 2023), which favours the best-case scenario with the median EDI 140 ng/kg of b.w./day. However, two major uncertainties with respect to BT exposure and pharmacokinetics need to be considered. Firstly, due to unknown pharmacokinetics, the estimated daily intake model based on urinary levels of free or conjugated forms of BTs cannot account for the quick degradation of BTs in the body after ring opening and the formation of ring scission products. Hence, the risk of underestimation is higher in the best-case scenario compared to other scenarios because it assumes that 90% of BT intake is excreted via urine in free or conjugated forms. Secondly, dietary exposure might be a significant source of BTs for humans, due to the contamination of aquatic ecosystems and the irrigation of the agricultural fields with contaminated water. However, only limited information is available regarding this exposure pathway for humans (Castro et al. 2023a, b; LeFevre et al. 2017). Moreover, the available human exposure studies didn´t measure 1M-BTR, which, as we suggested above, should be considered due to its presence in the environment as a potential product of microbial degradation (Alotaibi et al. 2015; Huntscha et al. 2014; Liu et al. 2011).

One of the strengths of the current study is that it fills a gap in understanding of BT exposure profiles in Czech men, with a special focus on firefighters. In particular, it sheds new light on exposure predictors as well as the associations with liver function (5 biomarkers), serum lipids (2 biomarkers), and oxidative stress (1 biomarker). In fact, this is the first study of its size in Europe, providing valuable insights into BT exposure in this region and demographic group. Furthermore, we report the levels of 1M-BTR in human urine for the first time and suggest the use of this compound for human biomonitoring.

In terms of limitations, the relatively small sample size and homogeneity of the study population need to be taken into account, because they determine the statistical strength of the tests and the ability to draw firm conclusions as well as to make generalisations about the whole Czech population. Although the models presented were controlled for confounding factors, residual bias by unmeasured factors cannot be excluded. Single spot urine samples were used for analyses, which, due to unknown pharmacokinetics, can potentially lead to additional variability in the measured concentrations. Lastly, the employed biomarkers of liver function and serum lipids are used as clinical biomarkers of deteriorating cardiovascular conditions; hence, the use of more sensitive biomarkers would probably be better for the exploration of potential adverse effects of BTs in the healthy and physically active male population.

## Conclusion

In conclusion, 6 BTRs and 2 BTHs were measured in 165 male urine samples from Czechia, the results providing the first insights into BT exposure in central Europe. Exposure to BTs was found to be highly prevalent among study population, and 1M-BTR was suggested as a new biomarker of BT exposure due to its high abundance in both urine as well as the environment. Within this study, associations between exposure to BTs and biomarkers of liver function, serum lipids, and oxidative stress were also assessed for the first time and the analyses did not reveal any strong associations. This work provides the basis for a deeper understanding of human BT exposure in Europe and its associations with liver function. Additional studies are warranted, especially in the areas of pharmacokinetics of BTs, dietary exposure, and effects on human health.

### Supplementary Information

Below is the link to the electronic supplementary material.

Supplementary file1 (DOCX 299 KB)

## Data Availability

The data generated and analysed during this study, including individual health, lifestyle, and chemical concentration data, are not publicly available due to sensitivity reasons. However, the data can be made available upon reasonable request to the corresponding author, subject to the establishment of data-sharing agreements. The data are securely stored in controlled access data storage at RECETOX, Brno, Czechia.
