# Supplementary material for: Exposure to benzotriazoles and benzothiazoles in Czech male population and its associations with biomarkers of liver function, serum lipids and oxidative stress
Source: Int Arch Occup Environ Health. 2024 Mar 28;97(5):523–36. doi: 10.1007/s00420-024-02059-x (PMC11130049; doi:10.1007/s00420-024-02059-x)
Supplement: Supplementary file 1 — Supplementary file1 (DOCX 299 KB) [file 420_2024_2059_MOESM1_ESM.docx]

Supplementary information:

**Exposure to benzotriazoles and benzothiazoles in Czech male population and its associations with biomarkers of liver function, serum lipids and oxidative stress**

**Journal:** International Archives of Occupational and Environmental Health

**Authors:** Nina Pálešová^a^, Lucie Bláhová^a^, Tomáš Janoš^a^, Katarína Řiháčková^a^, Aleš Pindur^a, b, c^, Ludmila Šebejová^a^, Pavel Čupr ^a^ (*)

^a^ RECETOX, Faculty of Science, Masaryk University, Kamenice 753/5, 625 00 Brno, Czech Republic

*^b^ Faculty of Sports Studies, Masaryk University, Kamenice 753/5, 625 00, Czech Republic*

*^c^ Training Centre of Fire Rescue Service, General Directorate of Fire Rescue Service of the Czech Republic, Ministry of the Interior, Trnkova 85, 628 00 Brno, Czech Republic*

(*) **corresponding author:** Pavel Čupr, RECETOX, Faculty of Science, Masaryk University, Kamenice 753/5, 624 00 Brno, Czech Republic, [pavel.cupr@recetox.muni.cz](mailto:pavel.cupr@recetox.muni.cz)

**Sample collection and storage**

Blood samples were collected by medical personnel in an operational ambulance. Urine samples were collected at the workplace by own urine collection following the instruction of medical personnel. In phases 1 and 3, morning void midstream urine was sampled, along with venous blood on an empty stomach. In phase 2, the sampling of morning void urine and venous blood on an empty stomach was not possible due to training schedule.

Venous blood for serum isolation was sampled in 7.5 mL S-Monovette® tube containing the Z-gel clotting activator. Each participant provided approximately 40 mL of midstream urine, which was collected in a 50 mL centrifuge tube. Both the venous blood and urine samples were immediately transported to laboratories in a cooling box set at 8°C.

Once the clot had formed in the venous blood tube, it was centrifuged at 2500×g and 20°C for 10 minutes. Subsequently, 0.5 mL aliquots were separated and placed into 1.2 mL cryotubes, which were then gradually frozen and stored in a biobank facility at -80°C for further biomarker and biochemical analyses. Similarly, the urine samples in 50 mL centrifuge tubes were divided into 1 mL aliquots in 1.2 mL cryotubes, frozen gradually, and stored in a biobank facility at -80°C until further analyses.

**Table S1 –** Results from the questionnaires (Řiháčková et al. 2023).

|  |  | **NEW** | **PROF** | **CTRL** |
| --- | --- | --- | --- | --- |
| **Participants that filled out questionnaires** |  | 59 | 52 | 55 |
| **Age (years)** | Median | 24.5 | 28 | 26 |
|  | 10th - 90th perc. | 21 - 31 | 23 - 33 | 20 - 32 |
|  | Min. - Max. | 19 - 34 | 20 - 35 | 18 - 35 |
| **BMI (kg/m^2^)** | Median | 26.3 | 26.2 | 24.6 |
|  | 10th - 90th perc. | 22.6 - 30.3 | 22.9 - 29 | 21.6 - 28.6 |
|  | Min. - Max. | 20.7 - 33.4 | 21.1 - 32.2 | 18.4 - 30.9 |
| **Infectious or chronic disease (%)** | Yes | 0 | 0 | 5.5 |
|  | No | 100 | 100 | 94.6 |
| **Health (subjective assessment, %)** | Always healthy and well | 50.9 | 67.3 | 56.4 |
|  | Mostly healthy and well | 49.2 | 32.7 | 41.8 |
|  | Often do not feel well | 0 | 0 | 1.8 |
| **Job (%)** | Firefighter | 100 | 100 | 0 |
|  | Student | 0 | 0 | 45.5 |
|  | IT | 0 | 0 | 14.6 |
|  | Office | 0 | 0 | 12.7 |
|  | Other | 0 | 0 | 27.2 |
| **Length of firefighting career (years)** | Median | 0.5 | 3.3 | 0 |
|  | 10th - 90th perc. | 0.25 - 1 | 1 - 10 | 0 |
|  | Min. - Max. | 0 - 5 | 0.5 - 14 | 0 |
| **Smoking (%)** | Yes | 0 | 0 | 0 |
|  | No | 100 | 100 | 100 |
| **Former smoking (%)** | Yes | 11.9 | 19.2 | 7.3 |
|  | No | 88.1 | 80.8 | 92.7 |
| **If a former smoker (years since quitting)** | Median | 4 | 0.5 | 3.5 |
|  | 10th - 90th perc. | 0.5 – 10 | 0.2 - 5 | 0.6 – 8 |
|  | Min. - Max | 0.5 - 10 | 0.1 - 6 | 0.6 - 8 |
| **Contact with a large fire in the last 6 months (%)** | Two or more times | 22 | 59.6 | 5.5 |
|  | One time | 32.2 | 21.2 | 0 |
|  | Never | 45.8 | 19.2 | 90.9 |
| **Contact with firefighting foams in the last year (%)** | Two or more times | 0 | 34.6 | 0 |
|  | One time | 25.4 | 40.4 | 1.8 |
|  | Never | 69.5 | 25 | 98.2 |
| **Diet (%)** | Mixed diet | 100 | 100 | 100 |
|  | Vegetarian | 0 | 0 | 0 |
|  | Vegan | 0 | 0 | 0 |
| **Use of food supplements (%)** | Yes | 27.1 | 17.3 | 56.4 |
|  | No | 72.9 | 82.7 | 43.6 |

**Chemicals and Reagents**

Methanol (99.95%), isopropanol (99.9%) and ammonium acetate (99.0%) were purchased from Biosolve Chimie (Dieuze, France), Honeywell (Seelze, Germany) and Sigma Aldrich (St. Louis, USA), respectively.

1-H-benzotriazole (1H-BTR, 98%) and 2-hydoxy-benzothiazole (2OH-BTH, 98%) were purchased from Sigma-Aldrich (St.Louis, USA). 1-methyl-benzotriazole (1M-BTR, 98%), 2-methyl-thio-benzothiazole (2MeS-BTH, 98%) and 2-amino-benzothiazole (2NH_2_-BTH, 98%) were purchased from Tokyo Chemical Industry Co., Ltd (Tokyo, Japan). 4-methyl-benzotriazole (98.5%) and 5-methyl-benzotriazole (98%) were purchased from Apollo Scientific Ltd (Cheshire, UK) and Alfa Aesar GmbH & Co KG (Karlsruhe, Germany), respectively. Xylyltriazole (XTR, 5,6-dimethyl-benzotriazole, 99%) was also purchased from Alfa Aesar GmbH & Co KG (Karlsruhe, Germany). 4-OH-benzotriazole (4OH-BTR, 95%) was purchased from Carbosynth Ltd (Compton, UK). Isotopically labelled standards d4-1-H-benzotriazole (d4-BTR) and atrazine-d5 were purchased from Toronto Research Chemicals (Toronto, Canada) and LGC Standards Ltd (Teddington, UK). β-glucuronidase from *Helix pomatia* (type HP-2, ≥100,000 units/mL β-glucuronidase, ≤7,500 units/mL sulfatase) was also purchased from Sigma-Aldrich.

**Table S2 –** Chemical characteristics and HPLC-MS/MS parameters for analysis BTs in urine.

| **Compound** | **MW** | **CAS** | **Parental ion m/z** | **Quantification/ Qualification ion m/z** | **MLOQ [μg/L]** | **RT [min]** | **IS** |
| --- | --- | --- | --- | --- | --- | --- | --- |
|  |  |  |  |  |  |  |  |
| 1H-BTR | 119.1 | 95-14-7 | 120.2 | **65.2/102.1** | 0.42 | 2.78 | d4-BTR |
| 1M-BTR | 133.1 | 13351-73-0 | 134.1 | **77.1/106.1/91.0** | 0.1 | 3.23 | d4-BTR |
| 4,5M-BTR | 133.1 | 29878-31-7;  136-85-6 | 134.1 | **77.2/79.1/106.2** | 0.98 | 3.37 | d4-BTR |
| 4OH-BTR | 135.1 | 26725-51-9 | 136.1 | **80.1/63.2/53.2** | 0.1 | 1.98 | d4-BTR |
| XTR | 147.2 | 4184-79-6 | 148.2 | **77.1/91.1/120.2** | 0.05 | 3.77 | d5-Atrazine |
| 2OH-BTH | 151.2 | 934-34-9 | 152.1 | **124.2/92.1/109.1** | 0.38 | 3.81 | d4-BTR |
| 2-NH2-BTH | 150.2 | 136-95-8 | 151.1 | **109.1/92.1/124.1** | 0.07 | 2.15 | d4-BTR |
| d4-1H-BTR | 123.2 | 1185072-03-0 | 124.2 | **69.2/96.2** | - | 2.76 | - |
| d5-atrazine | 220.7 | 163165-75-1 | 221.2 | **179.1/137.2** | - | 4.65 | - |

**Table S3** – Formulas used for correction of urine BT levels for urine dilution (Sauvé et al. 2015).

| Adjustment of BT levels for creatinine was performed by following formula:  $c_{{BTs}_{creat}}=\frac{c_{BTs}}{c_{creat}}$  where c_BTs_screat_ is creatinine adjusted BTs urine concentration [µg_BTs_ /g_creatinine_], c_BTs_ is BTs concentration in urine [ng/ml], and c_creat_ is urine creatinine concentration [g/l]. |
| --- |
| SG-corrected concentrations were calculated using following formula:  $C_{SG}= \frac{C_{i}\times({SG}_{ref}-1)}{{SG}_{meas}-1)}$  where C_SG_ is the concentration standardized on SG, Ci is the measured concentration, SG_meas_ is the measured specific gravity, and SG_ref_ is the reference SG value. As a reference SG value, a mean SG value for all cohorts was used (1.02). |

**Table S4 –** Recoveries from water (corrected to relevant internal standard) and urine (without correction) and concentrations of analytes in procedural blanks (n=8). 1 – mean recovery and RDS (%), spike 0.5, 5, 50, 160 ng/mL in water (n=4); 2 – mean recovery and RSD (%), spike 2 ng/mL for d5-atrazine and 10 ng/mL for d4-BTR (n=10)).

| **Compound** | **Recovery** | | **Blank concentrations** | |
| --- | --- | --- | --- | --- |
|  | **From water (%) 1** | **From urine (%) 2** | **Mean ± RSD %** | **Median** |
| 1H-BTR | 116.3 ± 4.5 % | - | 0.77 ± 18.4 | 0.82 |
| 1M-BTR | 97.4 ± 8.5 % | - | - | - |
| 4,5M-BTR | 101.6 ± 1.5 % | - | 1.57 ± 20.8 | 1.67 |
| 4OH-BTR | 110.7 ± 9.7 % | - | - | - |
| XTR | 94.4 ± 19 % | - | - | - |
| 2OH-BTH | 109.1 ± 9.3 % | - | 1.17 ± 10.8 | 1.16 |
| 2-NH2-BTH | 107.4 ± 9.9 % | - | 0.27 ± 8 | 0.27 |
| d4-BTR | - | 32.5 ± 23.9 % | - | - |
| d5-atrazine | - | 33.6 ± 28.8 % | - | - |

**Fig. S1** – Directed acyclic graph (Shrier and Platt 2008) for multiple linear regression model. BTs – benzotriazoles and benzothiazoles.


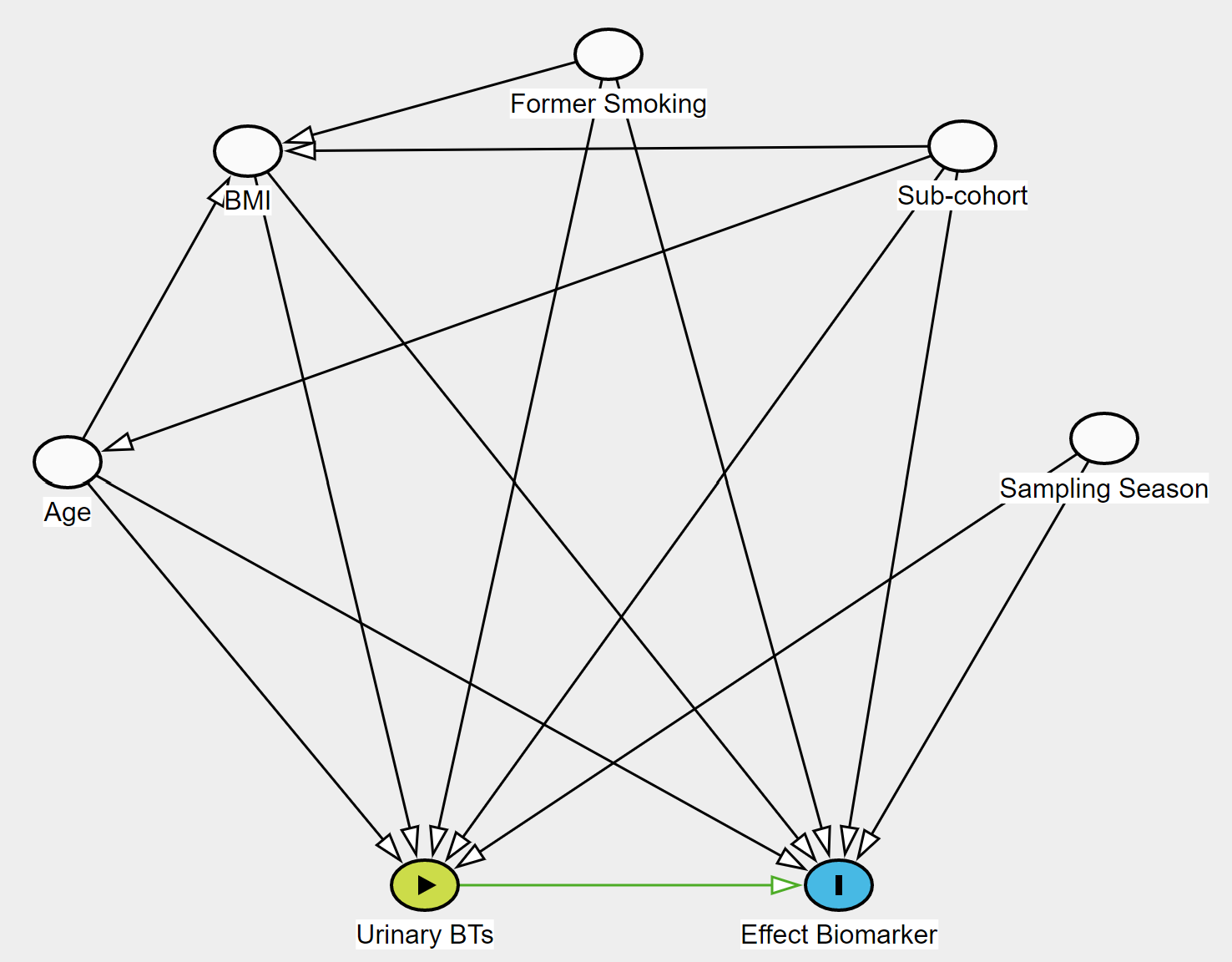


**Figure S2** – Spearman correlation matrix. Statistically significant correlations (p<0.05) are marked with “*”.


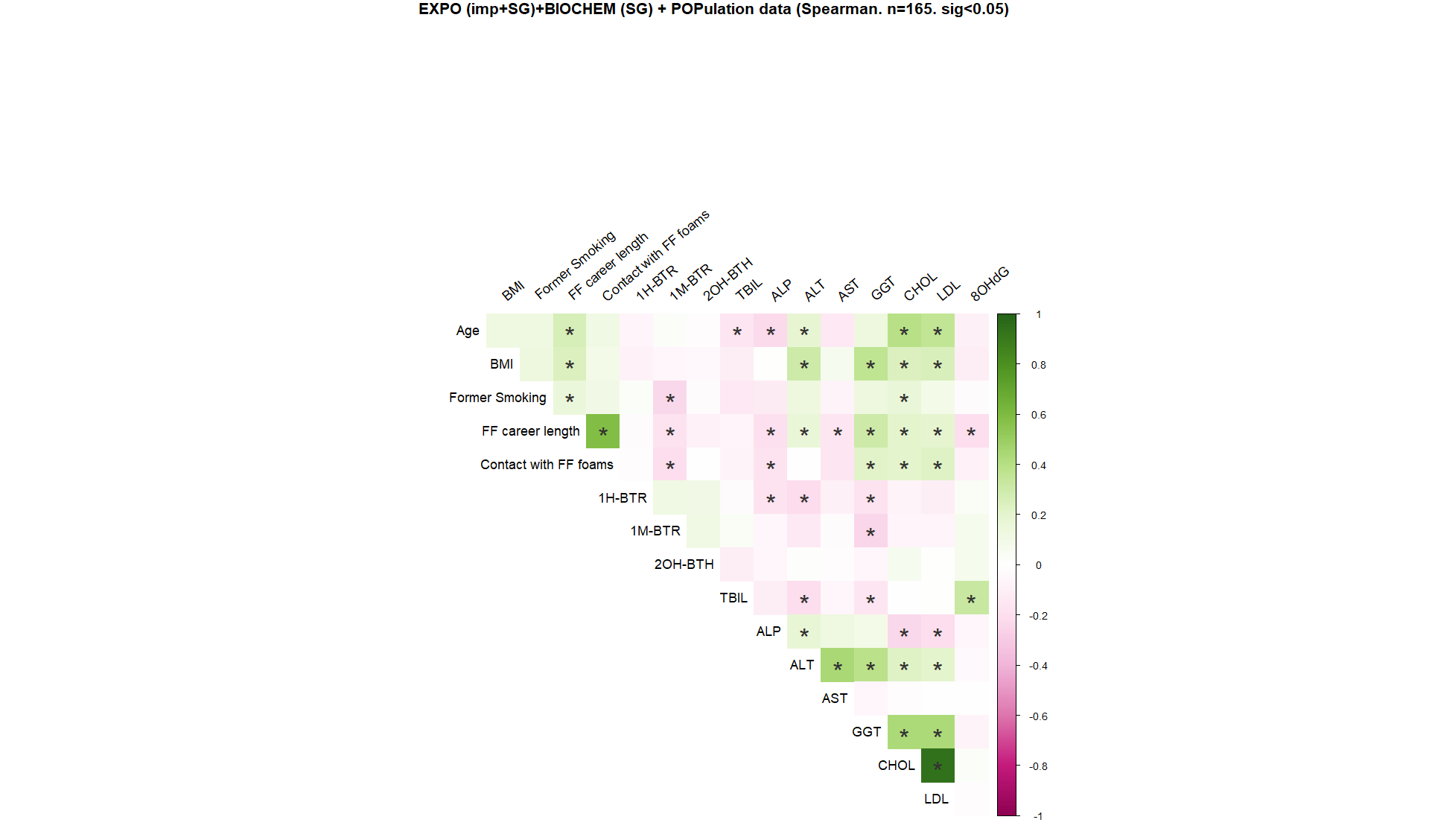


**Table S5** – Distribution profiles of creatinine-adjusted urinary BT concentrations (µg/g of creatinine) in complete dataset of CELSPAC-FIREexpo (n=165) as well as in the study sub-cohorts.
“ * “ – statistically different from professional firefighters (p<0.05).

| **Complete CELSPAC-FIREexpo dataset (n = 165)** | | | | | | | |  |
| --- | --- | --- | --- | --- | --- | --- | --- | --- |
|  | **Mean** | **Sd** | **Min** | **Pctile [25]** | **Median** | **Pctile [75]** | **Max** |  |
| **1H-BTR** | 0.51 | 1.00 | <MLOQ | <MLOQ | 0.14 | 0.54 | 8.5 |  |
| **4-OH-BTR** | 0.24 | 0.89 | <MLOQ | <MLOQ | <MLOQ | <MLOQ | 6.5 |  |
| **1-M-BTR** | 2.30 | 4.10 | <MLOQ | 0.11 | 1.00 | 2.20 | 26.0 |  |
| **4/5-M-BTR** | <MLOQ |  | <MLOQ | <MLOQ | <MLOQ | <MLOQ | 0.67 |  |
| **XTR** | <MLOQ |  | <MLOQ | <MLOQ | <MLOQ | <MLOQ | 0.28 |  |
| **2-OH-BTH** | 1.50 | 1.50 | <MLOQ | 0.57 | 1.20 | 2.10 | 11.0 |  |
| **2-NH2-BTH** | <MLOQ |  | <MLOQ | 0.00 | 0.00 | 0.00 | 1.0 |  |
| **Controls (n = 55)** | | | | | | | |  |
|  | **Mean** | **Sd** | **Min** | **Pctile [25]** | **Median** | **Pctile [75]** | **Max** |  |
| **1H-BTR** | 0.48 | 0.84 | <MLOQ | <MLOQ | 0.28 | 0.52 | 5.2 |  |
| **4-OH-BTR** | 0.13 | 0.44 | <MLOQ | <MLOQ | <MLOQ | <MLOQ | 3.0 |  |
| **1-M-BTR** | 2.40 | 3.70 | <MLOQ | 0.68 | 1.20 | 2.20 | 22.0 |  |
| **4/5-M-BTR** | <MLOQ |  | <MLOQ | <MLOQ | <MLOQ | <MLOQ | <MLOQ |  |
| **XTR** | <MLOQ |  | <MLOQ | <MLOQ | <MLOQ | <MLOQ | 0.28 |  |
| **2-OH-BTH** | 1.60 | 1.90 | <MLOQ | 0.46 | 1.10 | 2.10 | 11.0 |  |
| **2-NH2-BTH** | <MLOQ |  | <MLOQ | <MLOQ | <MLOQ | <MLOQ | 0.08 |  |
| **New firefighters in training (n = 58)** | | | | | | | |  |
|  | **Mean** | **Sd** | **Min** | **Pctile [25]** | **Median** | **Pctile [75]** | **Max** |  |
| **1H-BTR** | 0.47 | 0.72 | <MLOQ | <MLOQ | 0.22 | 0.57 | 2.9 |  |
| **4-OH-BTR** | 0.30 | 1.10 | <MLOQ | <MLOQ | <MLOQ | <MLOQ | 6.5 |  |
| **1-M-BTR** | 2.20 | 3.60 | <MLOQ | <MLOQ | 1.0 | 2.5 | 18.0 |  |
| **4/5-M-BTR** | <MLOQ |  | <MLOQ | <MLOQ | <MLOQ | <MLOQ | 0.67 |  |
| **XTR** | <MLOQ |  | <MLOQ | <MLOQ | <MLOQ | <MLOQ | 0.09 |  |
| **2-OH-BTH** | 1.80 | 1.30 | <MLOQ | 0.98 | 1.4 | 2.4 | 6.6 | * |
| **2-NH2-BTH** | <MLOQ |  | <MLOQ | <MLOQ | <MLOQ | <MLOQ | 0.18 |  |
| **Professional firefighters (n = 52)** | | | | | | | |  |
|  | **Mean** | **Sd** | **Min** | **Pctile [25]** | **Median** | **Pctile [75]** | **Max** |  |
| **1H-BTR** | 0.58 | 1.40 | <MLOQ | <MLOQ | <MLOQ | 0.53 | 8.5 |  |
| **4-OH-BTR** | 0.28 | 0.96 | <MLOQ | <MLOQ | <MLOQ | <MLOQ | 5.4 |  |
| **1-M-BTR** | 2.40 | 4.90 | <MLOQ | <MLOQ | 0.73 | 1.6 | 26.0 |  |
| **4/5-M-BTR** | <MLOQ |  | <MLOQ | <MLOQ | <MLOQ | <MLOQ | <MLOQ |  |
| **XTR** | <MLOQ |  | <MLOQ | <MLOQ | <MLOQ | <MLOQ | 0.03 |  |
| **2-OH-BTH** | 1.10 | 1.10 | <MLOQ | 0.16 | 0.94 | 1.6 | 5.9 |  |
| **2-NH2-BTH** | <MLOQ |  | <MLOQ | <MLOQ | <MLOQ | <MLOQ | 1.0 |  |

**Table S6** – Distribution profiles of unadjusted urinary BT concentrations (ng/mL) in complete dataset of CELSPAC-FIREexpo (n=165) as well as in the study sub-cohorts.
“ * “ – statistically different from professional firefighters (p<0.05).

| **Complete CELSPAC-FIREexpo dataset (n = 165)** | | | | | | | |  | |
| --- | --- | --- | --- | --- | --- | --- | --- | --- | --- |
| **Variable** | **Mean** | **Sd** | **Min** | **Pctile[25]** | **Median** | **Pctile[75]** | **Max** |  |  |
| **1H-BTR** | 0.87 | 1.50 | <MLOQ | <MLOQ | 0.42 | 1.20 | 12.0 |  |  |
| **4-OH-BTR** | 0.35 | 1.20 | <MLOQ | <MLOQ | <MLOQ | <MLOQ | 8.7 |  |  |
| **1-M-BTR** | 4.00 | 8.10 | <MLOQ | 0.17 | 1.80 | 4.10 | 84.0 |  |  |
| **4/5-M-BTR** | <MLOQ |  | <MLOQ | <MLOQ | <MLOQ | <MLOQ | 2.20 |  |  |
| **XTR** | <MLOQ |  | <MLOQ | <MLOQ | <MLOQ | <MLOQ | 0.48 |  |  |
| **2-OH-BTH** | 2.90 | 3.50 | <MLOQ | 1.10 | 2.00 | 3.80 | 30.0 |  |  |
| **2-NH2-BTH** | <MLOQ |  | <MLOQ | <MLOQ | <MLOQ | <MLOQ | 3.3 |  |  |
| **Controls (n = 55)** | | | | | | | |  |  |
| **Variable** | **Mean** | **Sd** | **Min** | **Pctile[25]** | **Median** | **Pctile[75]** | **Max** |  |  |
| **1H-BTR** | 1.00 | 1.60 | <MLOQ | <MLOQ | 0.54 | 1.20 | 8.7 |  |  |
| **4-OH-BTR** | 0.23 | 0.65 | <MLOQ | <MLOQ | <MLOQ | <MLOQ | 3.8 |  |  |
| **1-M-BTR** | 4.90 | 6.10 | <MLOQ | 1.40 | 2.70 | 4.30 | 23.0 | * |  |
| **4/5-M-BTR** | <MLOQ |  | <MLOQ | <MLOQ | <MLOQ | <MLOQ | 0.00 |  |  |
| **XTR** | <MLOQ |  | <MLOQ | <MLOQ | <MLOQ | <MLOQ | 0.48 |  |  |
| **2-OH-BTH** | 3.60 | 4.60 | <MLOQ | 1.00 | 2.10 | 4.90 | 30.0 | * |  |
| **2-NH2-BTH** | <MLOQ |  | <MLOQ | <MLOQ | <MLOQ | <MLOQ | 0.2 |  |  |
| **New firefighters in training (n = 58)** | | | | | | | |  |  |
| **Variable** | **Mean** | **Sd** | **Min** | **Pctile[25]** | **Median** | **Pctile[75]** | **Max** |  |  |
| **1H-BTR** | 0.82 | 1.10 | <MLOQ | <MLOQ | 0.54 | 1.30 | 4.5 |  |  |
| **4-OH-BTR** | 0.44 | 1.50 | <MLOQ | <MLOQ | <MLOQ | <MLOQ | 8.7 |  |  |
| **1-M-BTR** | 3.40 | 5.10 | <MLOQ | <MLOQ | 1.90 | 4.50 | 29.0 |  |  |
| **4/5-M-BTR** | <MLOQ |  | <MLOQ | <MLOQ | <MLOQ | <MLOQ | 2.20 |  |  |
| **XTR** | <MLOQ |  | <MLOQ | <MLOQ | <MLOQ | <MLOQ | 0.12 |  |  |
| **2-OH-BTH** | 3.40 | 2.90 | <MLOQ | 1.60 | 2.90 | 4.50 | 18.0 | * |  |
| **2-NH2-BTH** | <MLOQ |  | <MLOQ | <MLOQ | <MLOQ | <MLOQ | 0.3 |  |  |
| **Professional firefighters (n = 52)** | | | | | | | |  |  |
| **Variable** | **Mean** | **Sd** | **Min** | **Pctile[25]** | **Median** | **Pctile[75]** | **Max** |  |  |
| **1H-BTR** | 0.79 | 1.90 | <MLOQ | <MLOQ | <MLOQ | 0.75 | 12.0 |  |  |
| **4-OH-BTR** | 0.37 | 1.40 | <MLOQ | <MLOQ | <MLOQ | <MLOQ | 7.6 |  |  |
| **1-M-BTR** | 3.80 | 12.00 | <MLOQ | <MLOQ | 0.91 | 2.20 | 84.0 |  |  |
| **4/5-M-BTR** | <MLOQ |  | <MLOQ | <MLOQ | <MLOQ | <MLOQ | <MLOQ |  |  |
| **XTR** | <MLOQ |  | <MLOQ | <MLOQ | <MLOQ | <MLOQ | 0.07 |  |  |
| **2-OH-BTH** | 1.70 | 1.90 | <MLOQ | 0.28 | 1.60 | 2.10 | 12.0 |  |  |
| **2-NH2-BTH** | <MLOQ |  | <MLOQ | <MLOQ | <MLOQ | <MLOQ | 3.3 |  |  |

**Table S7 -** Detection frequencies (DF, %) and distribution profiles of unadjusted, specific gravity-adjusted and creatinine-adjusted urinary BT concentrations (ng/mL) in subset (n=20) with repeated measurement after 10 weeks. “(SG)” – refers to adjusted by specific gravity; “(CR)” – refers to adjusted by creatine.

| **1st measurement (week 1)** | | | | | | | | |
| --- | --- | --- | --- | --- | --- | --- | --- | --- |
| **Variable** | **DF** | **Mean** | **Sd** | **Min** | **Pctile[25]** | **Median** | **Pctile[75]** | **Max** |
| **1H-BTR** | 65.00 | 0.76 | 0.80 | <MLOQ | <MLOQ | 0.57 | 1.1 | 2.90 |
| **1M-BTR** | 90.00 | 6.00 | 7.10 | <MLOQ | 1.70 | 3.20 | 7.6 | 23.00 |
| **2OH-BTH** | 50.00 |  |  | <MLOQ | <MLOQ | <MLOQ | 2.3 | 7.40 |
| **1H-BTR (SG)** | 65.00 | 0.72 | 0.89 | <MLOQ | <MLOQ | 0.50 | 0.97 | 3.50 |
| **1M-BTR (SG)** | 90.00 | 5.90 | 7.10 | <MLOQ | 1.20 | 3.10 | 6.50 | 21.00 |
| **2OH-BTH (SG)** | 50.00 |  |  | <MLOQ | <MLOQ | <MLOQ | 2.1 | 4.80 |
| **1H-BTR (CR)** | 65.00 | 0.44 | 0.67 | <MLOQ | <MLOQ | 0.30 | 0.5 | 2.90 |
| **1M-BTR (CR)** | 90.00 | 3.70 | 4.30 | <MLOQ | 0.91 | 2.0 | 4.80 | 15.00 |
| **2OH-BTH (CR)** | 50.00 |  |  | <MLOQ | <MLOQ | 0.1 | 1.00 | 3.20 |
| **2nd measurement (week 10)** | | | | | | | | |
| **Variable** | **DF** | **Mean** | **Sd** | **Min** | **Pctile[25]** | **Median** | **Pctile[75]** | **Max** |
| **1H-BTR** | 50.00 |  |  | <MLOQ | <MLOQ | <MLOQ | 0.9 | 4.20 |
| **1M-BTR** | 85.00 | 4.50 | 5.80 | <MLOQ | 0.31 | 2.60 | 5.5 | 20.00 |
| **2OH-BTH** | 45.00 |  |  | <MLOQ | <MLOQ | <MLOQ | 1.2 | 3.90 |
| **1H-BTR (SG)** | 50.00 |  |  | <MLOQ | <MLOQ | <MLOQ | 0.86 | 4.20 |
| **1M-BTR (SG)** | 85.00 | 4.70 | 7.50 | <MLOQ | 0.42 | 2.10 | 6.80 | 33.00 |
| **2OH-BTH (SG)** | 45.00 |  |  | <MLOQ | <MLOQ | <MLOQ | 0.9 | 3.50 |
| **1H-BTR (CR)** | 50.00 |  |  | <MLOQ | <MLOQ | <MLOQ | 0.5 | 2.40 |
| **1M-BTR (CR)** | 85.00 | 3.70 | 7.10 | <MLOQ | 0.24 | 0.8 | 4.50 | 32.00 |
| **2OH-BTH (CR)** | 45.00 |  |  | <MLOQ | <MLOQ | <MLOQ | 0.44 | 2.30 |

**Table S8** – Detection frequencies (DF, %) and distribution profiles of SG-adjusted urinary BT concentrations (ng/mL) in sub-cohorts of CELSPAC-FIREexpo study (n=165). “ * “ – statistically different from professional firefighters (p<0.05).

|  | **Controls (n = 55)** | | | | | | | | | |
| --- | --- | --- | --- | --- | --- | --- | --- | --- | --- | --- |
|  | | **DF** | **Mean** | **SD** | **Min** | **Pctile[25]** | **Median** | **Pctile[75]** | **Max** |  |
| **1H-BTR** | | 58.2 | 0.90 | 1.54 | <MLOQ | <MLOQ | 0.49 | 0.89 | 8.42 |  |
| **4-OH-BTR** | | 18.2 | 0.21 | 0.66 | <MLOQ | <MLOQ | <MLOQ | <MLOQ | 4.29 |  |
| **1-M-BTR** | | 89.1 | 4.33 | 6.03 | <MLOQ | 1.20 | 2.22 | 4.01 | 34.18 | * |
| **4/5-M-BTR** | | 0.0 | <MLOQ |  | <MLOQ | <MLOQ | <MLOQ | <MLOQ | 0.00 |  |
| **XTR** | | 7.3 | <MLOQ |  | <MLOQ | <MLOQ | <MLOQ | <MLOQ | 0.43 |  |
| **2-OH-BTH** | | 83.6 | 2.92 | 3.44 | <MLOQ | 0.88 | 1.83 | 4.44 | 20.99 |  |
| **2-NH2-BTH** | | 3.6 | <MLOQ |  | <MLOQ | <MLOQ | <MLOQ | <MLOQ | 0.22 |  |
|  | **New firefighters in training (n = 58)** | | | | | | | | | |
|  | | **DF** | **Mean** | **SD** | **Min** | **Pctile[25]** | **Median** | **Pctile[75]** | **Max** |  |
| **1H-BTR** | | 56.9 | 0.75 | 1.08 | <MLOQ | <MLOQ | 0.44 | 1.03 | 5.36 |  |
| **4-OH-BTR** | | 19.0 | 0.48 | 1.85 | <MLOQ | <MLOQ | <MLOQ | <MLOQ | 12.09 |  |
| **1-M-BTR** | | 70.7 | 3.54 | 5.85 | <MLOQ | <MLOQ | 1.76 | 4.20 | 30.01 |  |
| **4/5-M-BTR** | | 5.2 | <MLOQ |  | <MLOQ | <MLOQ | <MLOQ | <MLOQ | 1.95 |  |
| **XTR** | | 5.2 | <MLOQ |  | <MLOQ | <MLOQ | <MLOQ | <MLOQ | 0.13 |  |
| **2-OH-BTH** | | 89.7 | 2.91 | 2.37 | <MLOQ | 1.68 | 2.28 | 4.11 | 15.70 | * |
| **2-NH2-BTH** | | 3.4 | <MLOQ |  | <MLOQ | <MLOQ | <MLOQ | <MLOQ | 0.22 |  |
|  | **Professional firefighters (n = 52)** | | | | | | | | | |
|  | | **DF** | **Mean** | **SD** | **Min** | **Pctile[25]** | **Median** | **Pctile[75]** | **Max** |  |
| **1H-BTR** | | 34.6 | 0.86 | 2.17 | <MLOQ | <MLOQ | <MLOQ | 0.88 | 13.21 |  |
| **4-OH-BTR** | | 13.5 | 0.41 | 1.47 | <MLOQ | <MLOQ | <MLOQ | <MLOQ | 8.91 |  |
| **1-M-BTR** | | 71.2 | 3.24 | 7.15 | <MLOQ | <MLOQ | 1.14 | 2.55 | 46.79 |  |
| **4/5-M-BTR** | | 0.0 | <MLOQ |  | <MLOQ | <MLOQ | <MLOQ | <MLOQ | 0.00 |  |
| **XTR** | | 1.9 | <MLOQ |  | <MLOQ | <MLOQ | <MLOQ | <MLOQ | 0.05 |  |
| **2-OH-BTH** | | 75.0 | 1.65 | 1.62 | <MLOQ | 0.26 | 1.45 | 2.17 | 8.64 |  |
| **2-NH2-BTH** | | 1.9 | <MLOQ |  | <MLOQ | <MLOQ | <MLOQ | <MLOQ | 1.82 |  |

**Table S9** – Results from linear regression performed as sensitivity analysis. Associations between creatinine-adjusted concentrations of BTs in urine and population characteristics. β-coefficient refers to relative change (%) in urinary BTs for unit increase/change in population characteristic. **Bold** indicates p-value < 0.05.

| **Characteristics** | **1H-BTR** | | **1M-BTR** | | **2OH-BTH** | |
| --- | --- | --- | --- | --- | --- | --- |
|  | **β** | **p-value** | **β** | **p-value** | **β** | **p-value** |
| **Age** | 2.8 | 0.819 | 8.1 | 0.414 | 6.4 | 0.525 |
| **BMI** | -11.5 | 0.222 | 0.7 | 0.933 | -4.3 | 0.594 |
| **Sub-cohort** |  |  |  |  |  |  |
| CTRL | reference | | reference | | reference | |
| NEW FF | 11.4 | 0.539 | -22.0 | 0.074 | 35.5 | **0.032** |
| PROF | -26.2 | **0.035** | -26.2 | **0.035** | -9.4 | 0.495 |
| **Length of FF career** | -2.9 | 0.567 | -4.7 | 0.245 | -1.1 | 0.800 |
| **Contact with AFFFs in the last year** | | | | | | |
| Never | reference | | reference | | reference | |
| One time | 16.3 | 0.394 | -19.7 | 0.120 | 9.2 | 0.547 |
| Two or more times | -9.2 | 0.670 | -32.2 | **0.031** | -5.8 | 0.747 |
| **Sampling season** | |  |  |  |  |  |
| autumn | reference | | reference | | reference | |
| spring | 10.9 | 0.572 | -18.7 | 0.170 | -18.7 | 0.170 |
| summer | -1.4 | 0.937 | -21.7 | 0.091 | -21.7 | 0.091 |
| winter | -50.8 | 0.057 | -28.0 | 0.281 | -28.0 | 0.281 |
| **Former smoking** | |  |  |  |  |  |
| no | reference | | reference | | reference | |
| yes | 13.6 | 0.558 | -30.4 | **0.037** | -8.7 | 0.612 |

**Table S10** – Associations between urinary SG-adjusted BT concentrations and biomarkers of interest analysed by multiple linear regression in **Model 1** adjusted for age, BMI and former smoking. **Bold** indicates p-value < 0.05, **bold** **and *** indicates statistical significance after FDR correction.

| **Biomarker** | **BTs** | **β-coefficient** | **SE** | **P-value** | **95% CI** |
| --- | --- | --- | --- | --- | --- |
| **TBIL** | 1H-BTR | -1.392 | 4.559 | 0.754 | (-9.702 - 7.683) |
|  | 1M-BTR | 0.273 | 5.290 | 0.958 | (-9.432 - 11.019) |
|  | 2OH-BTH | -4.006 | 4.635 | 0.368 | (-12.221 - 4.979) |
| **ALP** | 1H-BTR | -13.244 | 5.490 | **0.009** | (-21.934 - -3.586) |
|  | 1M-BTR | -5.242 | 6.501 | 0.394 | (-16.326 - 7.309) |
|  | 2OH-BTH | -4.601 | 5.707 | 0.397 | (-14.505 - 6.45) |
| **ALT** | 1H-BTR | -10.909 | 5.099 | **0.021** | (-19.243 - -1.716) |
|  | 1M-BTR | -12.044 | 5.925 | **0.027** | (-21.495 - -1.456) |
|  | 2OH-BTH | 3.036 | 5.279 | 0.562 | (-6.918 - 14.054) |
| **AST** | 1H-BTR | -4.501 | 5.662 | 0.404 | (-14.342 - 6.472) |
|  | 1M-BTR | -2.150 | 6.585 | 0.734 | (-13.73 - 10.984) |
|  | 2OH-BTH | -3.095 | 5.777 | 0.576 | (-13.268 - 8.271) |
| **GGT** | 1H-BTR | -9.518 | 5.053 | **0.044** | (-17.912 - -0.266) |
|  | 1M-BTR | -17.543 | 5.724 | **0.001 *** | (-26.127 - -7.962) |
|  | 2OH-BTH | -1.562 | 5.215 | 0.757 | (-10.965 - 8.834) |
| **CHOL** | 1H-BTR | -3.892 | 4.869 | 0.405 | (-12.505 - 5.569) |
|  | 1M-BTR | -2.533 | 5.658 | 0.642 | (-12.572 - 8.658) |
|  | 2OH-BTH | 5.948 | 4.950 | 0.234 | (-3.694 - 16.555) |
| **LDL** | 1H-BTR | -6.214 | 4.734 | 0.167 | (-14.402 - 2.757) |
|  | 1M-BTR | -3.770 | 5.517 | 0.475 | (-13.453 - 6.997) |
|  | 2OH-BTH | 3.251 | 4.847 | 0.500 | (-5.963 - 13.367) |
| **8OHdG** | 1H-BTR | -1.026 | 4.632 | 0.820 | (-9.493 - 8.232) |
|  | 1M-BTR | 1.682 | 4.939 | 0.730 | (-7.554 - 11.84) |
|  | 2OH-BTH | 3.456 | 4.552 | 0.446 | (-5.252 - 12.964) |

**Table S11** – Associations between urinary SG-adjusted BT concentrations and biomarkers of interest analysed by multiple linear regression in **Model 2** adjusted for age, BMI, former smoking, sub-cohort and sampling season. **Bold** indicates p-value < 0.05, **bold** **and *** indicates statistical significance after FDR correction.

| **Biomarker** | **BTs** | **β-coefficient** | **SE** | **P-value** | **95% CI** |
| --- | --- | --- | --- | --- | --- |
| **TBIL** | 1H-BTR | -1.221 | 4.623 | 0.786 | (-9.658 - 8.003) |
|  | 1M-BTR | 0.608 | 5.398 | 0.908 | (-9.316 - 11.619) |
|  | 2OH-BTH | -4.808 | 4.765 | 0.291 | (-13.17 - 4.359) |
| **ALP** | 1H-BTR | -13.737 | 5.491 | **0.006** | (-22.382 - -4.129) |
|  | 1M-BTR | -7.461 | 6.544 | 0.223 | (-18.353 - 4.883) |
|  | 2OH-BTH | -7.034 | 5.790 | 0.197 | (-16.817 - 3.898) |
| **ALT** | 1H-BTR | -9.321 | 5.105 | 0.051 | (-17.815 - 0.051) |
|  | 1M-BTR | -11.991 | 5.942 | **0.028** | (-21.476 - -1.361) |
|  | 2OH-BTH | 5.814 | 5.326 | 0.278 | (-4.495 - 17.236) |
| **AST** | 1H-BTR | -3.484 | 5.700 | 0.523 | (-13.496 - 7.686) |
|  | 1M-BTR | -5.621 | 6.652 | 0.370 | (-16.895 - 7.182) |
|  | 2OH-BTH | -2.065 | 5.901 | 0.716 | (-12.553 - 9.68) |
| **GGT** | 1H-BTR | -8.340 | 4.973 | 0.075 | (-16.72 - 0.882) |
|  | 1M-BTR | -15.477 | 5.697 | **0.003** | (-24.24 - -5.7) |
|  | 2OH-BTH | 0.449 | 5.197 | 0.930 | (-9.118 - 11.024) |
| **CHOL** | 1H-BTR | -3.710 | 4.950 | 0.435 | (-12.475 - 5.934) |
|  | 1M-BTR | -2.275 | 5.789 | 0.683 | (-12.558 - 9.216) |
|  | 2OH-BTH | 8.889 | 5.081 | 0.088 | (-1.266 - 20.088) |
| **LDL** | 1H-BTR | -6.387 | 4.763 | 0.158 | (-14.609 - 2.626) |
|  | 1M-BTR | -3.814 | 5.589 | 0.476 | (-13.612 - 7.095) |
|  | 2OH-BTH | 6.408 | 4.932 | 0.199 | (-3.245 - 17.024) |
| **8OHdG** | 1H-BTR | 1.368 | 4.338 | 0.750 | (-6.79 - 10.239) |
|  | 1M-BTR | -1.725 | 4.680 | 0.704 | (-10.215 - 7.569) |
|  | 2OH-BTH | 5.272 | 4.273 | 0.221 | (-3.08 - 14.344) |

**Table S12** – Results from multiple linear regression performed as sensitivity analysis. Associations between urinary SG-adjusted BT concentrations and biomarkers of interest analysed by multiple linear regression in **multiple-BTs model** adjusted for all BTs, age, BMI, former smoking, sub-cohort, sampling season. **Bold** indicates p-value < 0.05.

| **Biomarker** | **BTs** | **β-coefficient** | **SE** | **P-value** | **95% CI** |
| --- | --- | --- | --- | --- | --- |
| **TBIL** | 1H-BTR | -0.755 | 4.713 | 0.870 | (-9.385 - 8.697) |
|  | 1M-BTR | 1.161 | 5.474 | 0.829 | (-8.948 - 12.392) |
|  | 2OH-BTH | -4.790 | 4.845 | 0.301 | (-13.286 - 4.539) |
| **ALP** | 1H-BTR | -12.672 | 5.588 | **0.014** | (-21.567 - -2.769) |
|  | 1M-BTR | -5.081 | 6.495 | 0.409 | (-16.177 - 7.484) |
|  | 2OH-BTH | -5.026 | 5.746 | 0.357 | (-14.95 - 6.057) |
| **ALT** | 1H-BTR | -8.939 | 5.118 | 0.063 | (-17.489 - 0.498) |
|  | 1M-BTR | -11.315 | 5.946 | **0.039** | (-20.879 - -0.596) |
|  | 2OH-BTH | 8.016 | 5.262 | 0.135 | (-2.39 - 19.532) |
| **AST** | 1H-BTR | -2.745 | 5.819 | 0.623 | (-13.027 - 8.752) |
|  | 1M-BTR | -5.102 | 6.764 | 0.425 | (-16.613 - 7.997) |
|  | 2OH-BTH | -1.350 | 5.983 | 0.815 | (-12.049 - 10.65) |
| **GGT** | 1H-BTR | -6.949 | 4.954 | 0.138 | (-15.426 - 2.377) |
|  | 1M-BTR | -14.721 | 5.755 | **0.005** | (-23.646 - -4.754) |
|  | 2OH-BTH | 2.527 | 5.093 | 0.616 | (-7.056 - 13.099) |
| **CHOL** | 1H-BTR | -4.541 | 5.006 | 0.343 | (-13.323 - 5.13) |
|  | 1M-BTR | -2.373 | 5.816 | 0.672 | (-12.69 - 9.162) |
|  | 2OH-BTH | 9.737 | 5.147 | 0.066 | (-0.622 - 21.175) |
| **LDL** | 1H-BTR | -6.868 | 4.832 | 0.134 | (-15.157 - 2.232) |
|  | 1M-BTR | -3.382 | 5.612 | 0.530 | (-13.263 - 7.624) |
|  | 2OH-BTH | 7.662 | 4.967 | 0.130 | (-2.171 - 18.483) |
| **8OHdG** | 1H-BTR | 0.953 | 4.405 | 0.826 | (-7.29 - 9.928) |
|  | 1M-BTR | -2.341 | 4.733 | 0.609 | (-10.868 - 7.002) |
|  | 2OH-BTH | 5.334 | 4.346 | 0.224 | (-3.158 - 14.57) |

**Table S13** – Results from multiple linear regression performed as sensitivity analysis. Associations between urinary creatinine-adjusted BT concentrations and biomarkers of interest analysed by multiple linear regression in Model 2 adjusted for age, BMI, former smoking, sub-cohort and sampling season. **Bold** indicates p-value < 0.05.

| **Biomarker** | **BTs** | **β-coefficient** | **SE** | **P-value** | **95% CI** |
| --- | --- | --- | --- | --- | --- |
| **TBIL** | 1H-BTR | -2.493 | 4.044 | 0.525 | (-9.838 - 5.451) |
|  | 1M-BTR | -0.488 | 5.139 | 0.922 | (-9.866 - 9.866) |
|  | 2OH-BTH | -7.388 | 4.979 | 0.116 | (-15.864 - 1.941) |
| **ALP** | 1H-BTR | -12.028 | 4.810 | **0.007** | (-19.824 - -3.473) |
|  | 1M-BTR | -7.092 | 6.228 | 0.225 | (-17.544 - 4.684) |
|  | 2OH-BTH | -7.568 | 6.077 | 0.184 | (-17.736 - 3.856) |
| **ALT** | 1H-BTR | -7.700 | 4.477 | 0.069 | (-15.35 - 0.641) |
|  | 1M-BTR | -11.098 | 5.662 | **0.034** | (-20.261 - -0.88) |
|  | 2OH-BTH | 7.068 | 5.584 | 0.211 | (-3.829 - 19.199) |
| **AST** | 1H-BTR | -3.090 | 4.989 | 0.520 | (-11.976 - 6.693) |
|  | 1M-BTR | -5.379 | 6.330 | 0.369 | (-16.182 - 6.817) |
|  | 2OH-BTH | -2.326 | 6.196 | 0.696 | (-13.262 - 9.989) |
| **GGT** | 1H-BTR | -8.282 | 4.342 | **0.044** | (-15.667 - -0.249) |
|  | 1M-BTR | -15.405 | 5.408 | **0.002** | (-23.764 - -6.129) |
|  | 2OH-BTH | -1.302 | 5.455 | 0.805 | (-11.133 - 9.617) |
| **CHOL** | 1H-BTR | -3.997 | 4.330 | 0.337 | (-11.709 - 4.388) |
|  | 1M-BTR | -2.728 | 5.508 | 0.607 | (-12.505 - 8.14) |
|  | 2OH-BTH | 8.165 | 5.345 | 0.134 | (-2.408 - 19.883) |
| **LDL** | 1H-BTR | -5.849 | 4.169 | 0.142 | (-13.148 - 2.063) |
|  | 1M-BTR | -3.834 | 5.319 | 0.452 | (-13.191 - 6.533) |
|  | 2OH-BTH | 6.370 | 5.180 | 0.223 | (-3.729 - 17.529) |
| **8OHdG** | 1H-BTR | 0.803 | 3.571 | 0.820 | (-5.948 - 8.037) |
|  | 1M-BTR | -1.608 | 3.867 | 0.670 | (-8.714 - 6.05) |
|  | 2OH-BTH | 0.971 | 3.507 | 0.779 | (-5.675 - 8.087) |

**Table S14** – Estimated daily intakes (µg/kg of body weight/day) for 3 theoretical toxicokinetic scenarios in 3 study sub-cohorts.

|  | **F_UE_** | **Mean** | **SD** | **Percentiles** | | |
| --- | --- | --- | --- | --- | --- | --- |
| **CTRL** |  |  |  | **25th** | **50th** | **75th** |
| Best-case scenario | 0.9 | 0.2 | 0.19 | 0.10 | 0.16 | 0.25 |
| Medium scenario | 0.5 | 0.4 | 0.34 | 0.18 | 0.28 | 0.44 |
| Worst-case scenario | 0.1 | 1.9 | 1.70 | 0.88 | 1.40 | 2.20 |
| **NEW** |  |  |  |  |  |  |
| Best-case scenario | 0.9 | 0.2 | 0.17 | 0.10 | 0.15 | 0.23 |
| Medium scenario | 0.5 | 0.4 | 0.30 | 0.18 | 0.27 | 0.41 |
| Worst-case scenario | 0.1 | 1.8 | 1.50 | 0.88 | 1.40 | 2.10 |
| **PROF** |  |  |  |  |  |  |
| Best-case scenario | 0.9 | 0.2 | 0.20 | 0.07 | 0.10 | 0.20 |
| Medium scenario | 0.5 | 0.3 | 0.36 | 0.13 | 0.18 | 0.36 |
| Worst-case scenario | 0.1 | 1.5 | 1.80 | 0.63 | 0.89 | 1.80 |

**References:**

Řiháčková, Katarína, Aleš Pindur, Klára Komprdová, Nina Pálešová, Jiří Kohoutek, Petr Šenk, Jana Navrátilová, Lenka Andrýsková, Ludmila Šebejová, Richard Hůlek, Mazen Ismael, and Pavel Čupr. 2023. “The Exposure of Czech Firefighters to Perfluoroalkyl Substances and Polycyclic Aromatic Hydrocarbons: CELSPAC – FIREexpo Case-Control Human Biomonitoring Study.” 881(April). doi: 10.1016/j.scitotenv.2023.163298.

Sauvé, Jean François, Martine Lévesque, Mélanie Huard, Daniel Drolet, Jérôme Lavoué, Robert Tardif, and Ginette Truchon. 2015. “Creatinine and Specific Gravity Normalization in Biological Monitoring of Occupational Exposures.” *Journal of Occupational and Environmental Hygiene* 12(2):123–29. doi: 10.1080/15459624.2014.955179.

Shrier, Ian, and Robert W. Platt. 2008. “Reducing Bias through Directed Acyclic Graphs.” *BMC Medical Research Methodology* 8:1–15. doi: 10.1186/1471-2288-8-70.
